# Beyond canonical asymmetric induction in phosphine organocatalysis

**DOI:** 10.1039/d6ra04151e

**Published:** 2026-07-06

**Authors:** Archana Vijayakumar, R. Bharath Krishna, M Manod, Adithya Appus, Chithra Mohan

**Affiliations:** a School of Chemical Sciences, Mahatma Gandhi University Kottayam 686560 India chithramohan84@mgu.ac.in; b The University of Texas at Dallas Richardson Texas 75080 USA rbharath.krishna@utdallas.edu.in; c Institute for Integrated Programmes and Research in Basic Sciences, Mahatma Gandhi University Kottayam 686560 India

## Abstract

Phosphine organocatalysis has emerged as a formidable and transformative platform for engineering reaction manifolds that depart from classical models of asymmetric induction, enabling cascade cyclizations to proceed through distinctive stereoelectronic and orbital interactions. Central to these transformations is the formation of fleeting, zwitterionic phosphonium ylides *via* nucleophilic addition of phosphines to electrophilic partners, which orchestrate bond formation with high stereocontrol under mild conditions. Even as phosphine catalysts have become central to asymmetric induction, a unified understanding of how they act as linchpins in choreographing non-canonical asymmetric induction during cascade cyclizations remains elusive, particularly in enabling the enantioselective construction of structurally diverse carbocyclic and heterocyclic frameworks. This work systematically maps phosphine-catalyzed enantioselective cascade cyclizations, organized by annulation topology of the reacting partners within their mechanistic pathways, and distils the design principles that govern chemo-, regio-, and stereoselectivity as well as reaction efficiency across multiple reaction manifolds. Furthermore, the study is structured as a comprehensive roadmap for the rational development of next-generation phosphine-catalyzed asymmetric transformations, linking mechanistic insight to synthetic strategy, and highlighting their expanding impact on organic synthesis. This Review addresses critical gaps in phosphine-catalyzed asymmetric annulation chemistry by integrating mechanistic and synthetic advances and identifying key unresolved challenges that will guide the development of next-generation stereoselective transformations.

## Abstract 2

This review deciphers the stereoelectronic logic underlying non-canonical asymmetric induction in phosphine-catalyzed cascade annulations.

## Graphical abstract

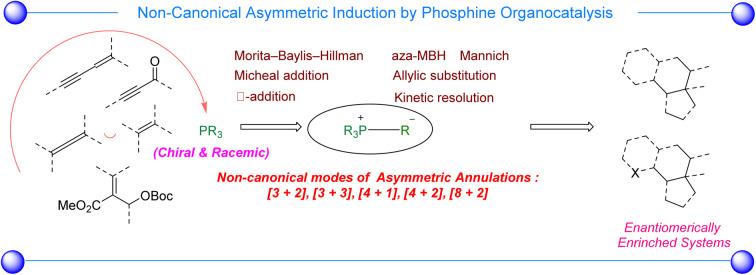

## 1. Introduction

Asymmetric organocatalysis has revolutionized synthetic methodology by delivering sustainable, atom-economic strategies that enable absolute stereochemical control in the construction of architecturally complex molecular frameworks.^1^ Various p-block elements, particularly phosphorus, nitrogen, and sulfur, constitute the cornerstone of modern organocatalyst design, owing to their ability to deliver finely tunable electronic and steric environments that govern reactivity and selectivity. Their intrinsic potential to function as Lewis bases or hydrogen-bond donors, coupled with exceptional compatibility under mild conditions, renders them indispensable for sustainable catalytic strategies.^2^ Over the past two decades, phosphine-based organic catalysts have emerged as pivotal players, accounting to their strong nucleophilicity, tunable electronic profiles, and the idiosyncratic capacity to generate zwitterionic intermediates in enabling diverse annulation pathways.^2,3^

In the realm of organophosphine chemistry, trialkyl- and arylphosphines were first recognized for their catalytic potential in the seminal hexamerization of acetonitrile.^4–7^ Despite landmark transformations such as the Rauhut–Currier and Morita–Baylis–Hillman (MBH) reactions,^8,9^ the intense potential of nucleophilic phosphine catalysis to orchestrate non-canonical asymmetric induction, during cascade cyclizations, delivering enantioenriched carbocyclic and heterocyclic frameworks with remarkable efficiency, remained largely untapped for decades. Non-canonical asymmetric induction involves non-classical modes of stereocontrol, where enantioselectivity is governed by non-covalent interactions, ion-pairing, and related secondary interactions rather than purely steric effects.

From the perspective of cascade cyclization, the seminal contribution by Lu *et al.* in 1995 marked a climacteric with the first phosphine-catalyzed [3 + 2] annulation of allenoates with electron-deficient olefins in the construction of cyclopentene derivatives.^10^ Since then, the phosphine-based organocatalysis has advanced relentlessly, with extensive catalyst tuning. Achiral phosphines such as triphenylphosphine have historically enabled numerous transformations, but the demand for enantioenriched products has driven the evolution toward chiral phosphine catalysts. Exceptional examples are mono-, multifunctional acyclic and cyclic chiral phosphines, investigated for their unprecedented stereoelectronic attributes that enable highly efficient asymmetric induction, thereby, cementing phosphine catalysis as a defining paradigm in contemporary asymmetric synthesis.^11–16^

Over the past decade, the advent of unconventional asymmetric induction mediated by phosphine catalysts has grabbed the attention of synthetic organic chemists, particularly for its transformative role in cascade cyclizations.^15^ In view of this surge of significant breakthroughs, several reviews have been addressed on phosphine organocatalysis, their scope has largely been remained confined to broad mechanistic overviews or specialized themes such as nucleophilic catalysis, strategies for mitigating phosphine oxide waste, and organophosphorus-mediated single-electron transfer processes.^17–21^ Yet, no review have systematically examined the pivotal role of phosphine catalysts as linchpins in enabling non-canonical asymmetric induction during cascade cyclizations – a game-changing methodological paradigm for the efficient construction of enantioenriched carbocyclic and heterocyclic molecular architectures central to modern synthetic design. This study aims to address this gap by providing a mechanistic analysis organized systematically by [3 + 2], [3 + 3], [4 + 1], [4 + 2], and miscellaneous annulation topology. Each mechanistic pathway is illustrated with its utility in constructing natural product skeletons and pharmacologically relevant scaffolds, highlighting the strategic role of phosphine catalysts in enabling stereoselective complexity generation.

This work offers the synthetic community a coherent framework on the capabilities of asymmetric phosphine organocatalysis, with particular emphasis on its ability to induce non-canonical asymmetric induction during cascade cyclizations. Analyzing mechanistic paradigms and illustrating their synthetic utility through representative examples in complex natural product synthesis, this work integrates organophosphine catalyst architectures, reactive ylide manifolds, stereochemical outcomes, and synthetic applications into a unified map of design principles. Throughout, we maintain a discussion that links ligand electronics to enantioselectivity, providing actionable insights for rational catalyst development. Building on these insights, this work will benefit the readers as it charts a roadmap for next-generation phosphine organocatalyst development – bridging fundamental mechanistic understanding with practical synthetic strategy, and underscoring the transformative role of phosphine organocatalysis in shaping the future of organic synthesis.

## 2. Asymmetric cascade reactions involving [3 + 2]-annulation

Since its seminal introduction by Lu and co-workers in 1995, phosphine-catalyzed [3 + 2] annulation, where a three atom synthon combines with a two atom synthon, has entered the core repertoire of synthetic methodology, profoundly enriching the construction of five-membered carbocyclic and heterocyclic frameworks.^22^ Maleimides are one of the extensively utilized C2 synthons in [3 + 2] reactions.^2,4^

**Scheme 1:**
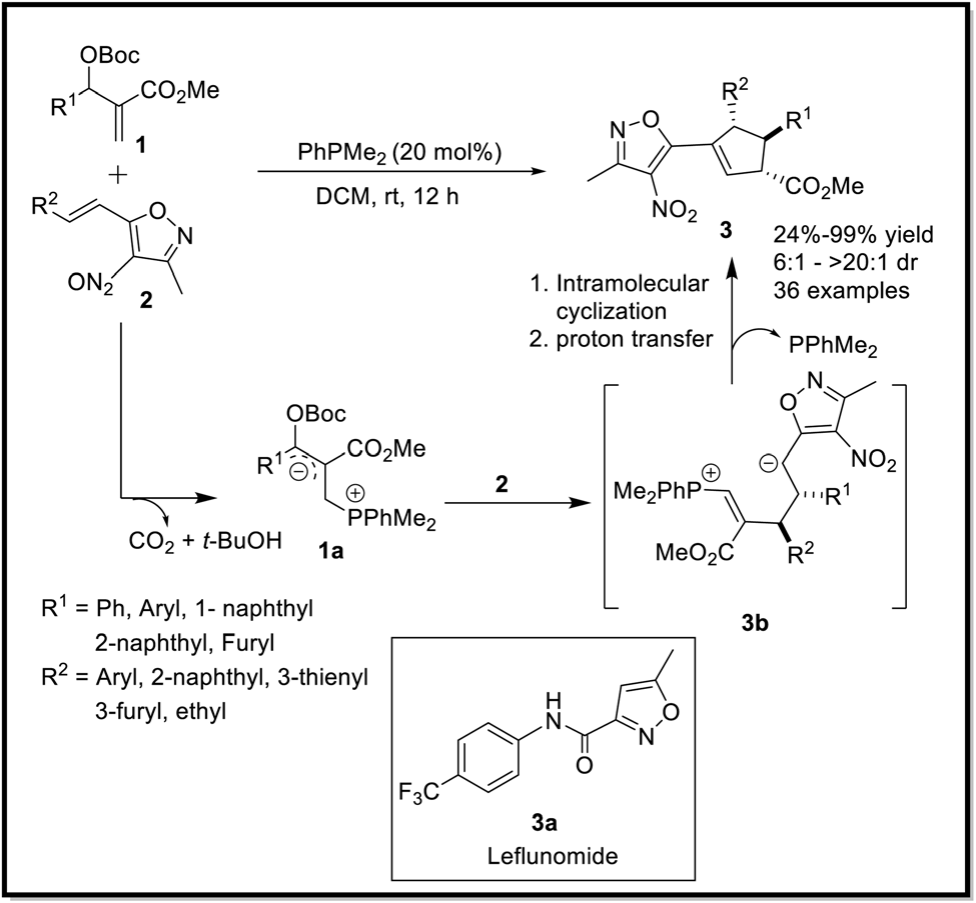
Cascade [3 + 2] annulation of MBH carbonates with nitrostyryl isoxazoles.

Although a broad range of MBH-derived electrophiles, including carbonates, halides, and acetates, have been explored in [3 + 2] annulation chemistry, recent efforts have converged on MBH carbonates, exemplified by the team of Liao *et al.* towards their PhPMe_2_ promoted phosphine-catalyzed annulation employing nitrostyrylisoxazoles as unconventional C2 synthons for the synthesis of structurally diverse isoxazole frameworks 3 (Scheme 1).^23^ A wide range of MBH carbonates 1 was accommodated, with steric effects exerting a pronounced influence on reactivity: *para*- and *meta*-substituted aryl derivatives afforded excellent yields, whereas *ortho*-substituted analogues displayed remarkably less efficiency; notably, the 1-naphthyl-substituted carbonate 1 proved unreactive, in contrast to its 2-naphthyl counterpart, which underwent smooth annulation. In comparison, aryl-substituted nitrostyrylisoxazoles 2 were largely insensitive to both positional and electronic variation. Mechanistically, nucleophilic addition of the phosphine to carbonate 1, followed by sequential extrusion of CO_2_ and *tert*-butanol, generates the zwitterionic ylide 1a, which undergoes regioselective addition to alkene 2 to form intermediate 3b; subsequent intramolecular cyclization, proton transfer, and catalyst elimination deliver isoxazoles 3 in good to excellent yields with high diastereoselectivity. The stereochemical outcome is governed by steric repulsion between the aryl substituents of 1 and 2, enforcing an *anti*-approach in intermediate 1b and culminating in a *trans* relationship between the phenyl and carboxyl substituents in 3. Notably, product 3 exhibits close structural resemblance to the bioactive isoxazole motif of leflunomide (3a), a clinically used dehydrogenase inhibitor, highlighting the potential of this annulation manifold for the modular synthesis of biologically relevant isoxazole-based architectures.

**Scheme 2:**
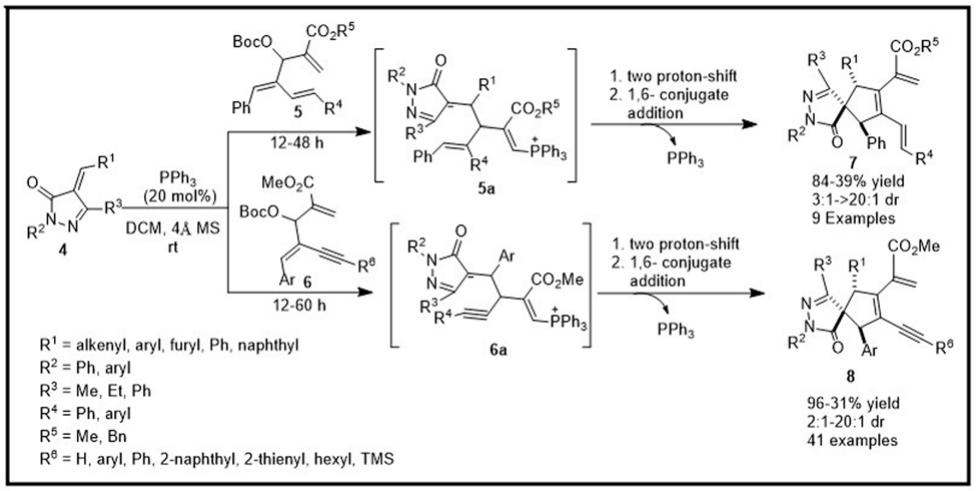
Cascade [3 + 2] annulation of γ-substituted Morita–Baylis–Hillman carbonates with exocyclic alkene.

Exocyclic olefins constitute privileged synthons for the construction of both fused and spirocyclic molecular architectures. Building on their earlier studies, Liao and co-workers in 2023 extended phosphine-catalyzed [3 + 2] annulation to cinnamaldehyde-derived MBH carbonates 5 and 6, which, upon reaction with exocyclic alkenes 4, delivered fully substituted spirocyclopentenes 7 and 8 with excellent diastereoselectivity (Scheme 2).^23^ Activation of γ-substituted MBH carbonates 5 or 6 by PPh_3_ enabled efficient annulation with exocyclic olefins 4, tolerating a broad array of alkenyl-, alkynyl-, and γ-hydrogen substituents and thereby allowing the formal incorporation of β-, γ-, and δ-carbon units into the spirocyclic products. Mechanistically, nucleophilic phosphine addition to carbonates 5 or 6, accompanied by sequential extrusion of CO_2_ and *tert*-butanol, generates zwitterionic ylides that undergo conjugate addition to alkene 4, forming intermediates 5a and 6a; subsequent dual proton-transfer events and intramolecular 1,6-conjugate addition, followed by phosphine elimination, furnish spirocyclopentenes 7 and 8 in high efficiency and stereochemical fidelity.

**Scheme 3:**
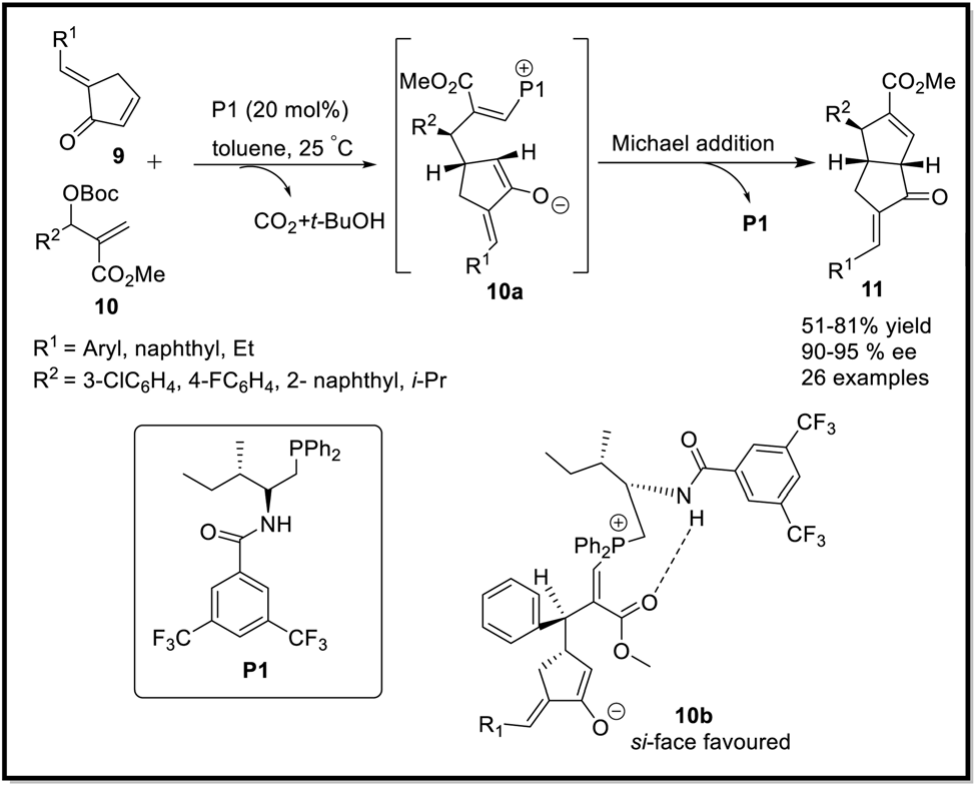
Cascade [3 + 2] annulation reaction of MBH-carbonates with cyclopent-2-enones.

By modifying this annulation paradigm to engage exocyclic cyclopentenes, Wang *et al.* developed an enantioselective phosphine-catalyzed [3 + 2] annulation between MBH carbonates 10 and cyclopent-2-enones 9, enabling access to fused cyclopentane architectures in the form of chiral hexahydropentalene derivatives 11 (Scheme 3).^24^ Enantioinduction is achieved through a chiral phosphine catalyst P1, which generates a stereodefined zwitterionic ylide upon nucleophilic addition to carbonate 10, followed by decarboxylation and *tert*-butanol elimination. The resulting ylide undergoes a facially selective *si*-face conjugate addition to cyclopentenone 9, forming enolate intermediate 10a, a selectivity governed by minimized steric interactions between the aryl and alkyl substituents of P1; subsequent intramolecular Michael addition and catalyst regeneration furnish product 11 with high enantiocontrol. Substrate effects reveal enhanced efficiency for *meta*-substituted aryl MBH carbonates 10 and *para*-substituted aryl cyclopentenones 9, with the protocol consistently delivering good yields and good-to-excellent enantioselectivities.

Beyond MBH carbonates, substituted allenes—particularly allenoates—have emerged as versatile electrophiles in phosphine-catalyzed annulation, enabling the efficient assembly of highly functionalized five-membered architectures.

**Scheme 4:**
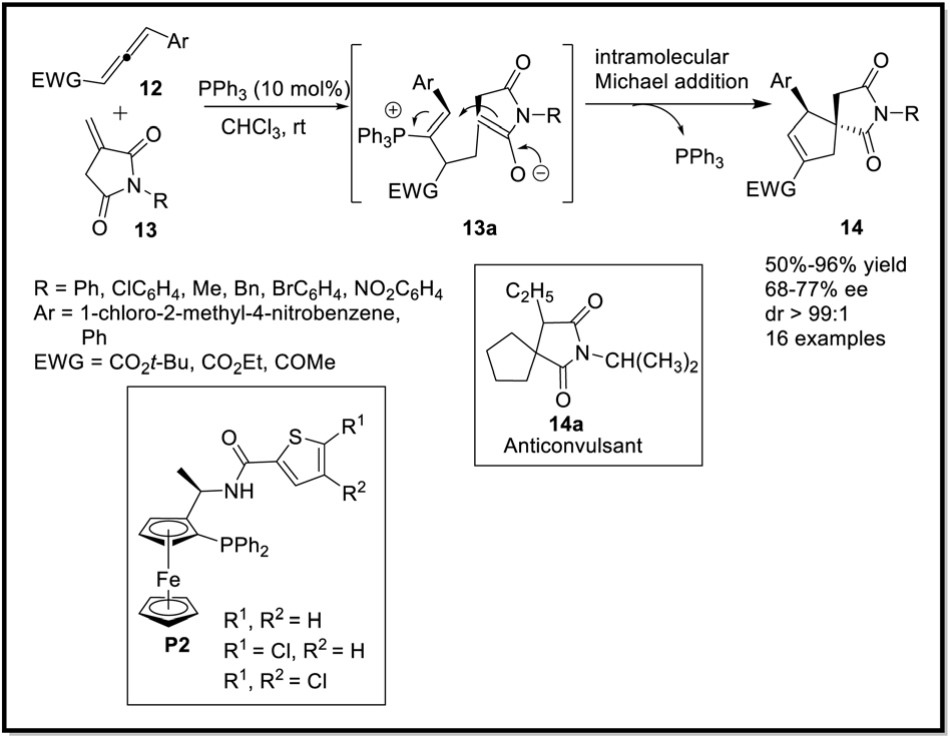
Cascade [3 + 2] annulation reaction of γ-substituted allenoates with *N*-methylidenesuccinimides.

In 2017, Luo and co-workers disclosed a phosphine-catalyzed [3 + 2] annulation of γ-substituted allenoates 12 with *N*-methylidenesuccinimides 13, delivering functional azaspirane derivatives 14 with excellent diastereo- and regioselectivity (dr > 99 : 1) (Scheme 4).^25^ Nucleophilic attack of triphenyl phosphine on allenoate 12 generates a zwitterionic intermediate that adds to succinimide 13, forming intermediate 13a, which undergoes intramolecular Michael addition and subsequent phosphine elimination to afford 14. The asymmetric variant, employing a bifunctional chiral ferrocenylphosphine P2, enabled the synthesis of 14 in moderate yields. Given the recognized anticonvulsant activity of azaspirane scaffolds, this phosphine-catalyzed strategy provides a modular and stereocontrolled route to biologically relevant azaspirane frameworks, exemplified by derivative 14a.

**Scheme 5:**
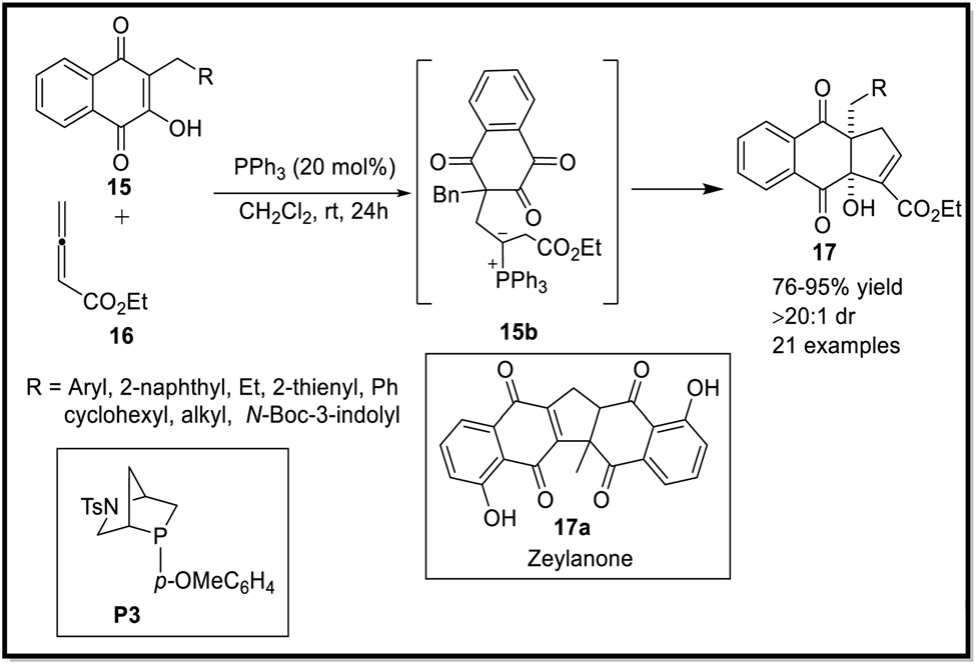
Phosphine-catalyzed [3 + 2] annulation reaction of allenoate with hydroxynaphthaquinone.

Building on their earlier work, the same group in 2018 expanded this diastereoselective phosphine-catalyzed strategy to construct diversely functionalized hydroxynaphthaquinones 17 using allenoate 16 and naphthaquinones 15 as C2 partners (Scheme 5).^26^ Substrate scope studies revealed broad tolerance, including electron-donating and -withdrawing aryl, naphthyl, and thiophenyl substituents, while alkyl-substituted naphthaquinones afforded products in good to excellent yields with excellent diastereoselectivity. Mechanistically, the transformation proceeds through an unprecedented γ-addition/aldol annulation pathway, delivering hydroxynaphthaquinones 17 structurally related to the bioactive natural product zeylanone (17a), a potent antibacterial, antifungal, and anticancer agent. This protocol thus provides a modular platform for accessing structurally complex naphthaquinone derivatives and could be extended toward the synthesis of a wider array of bioactive natural products.

**Scheme 6:**
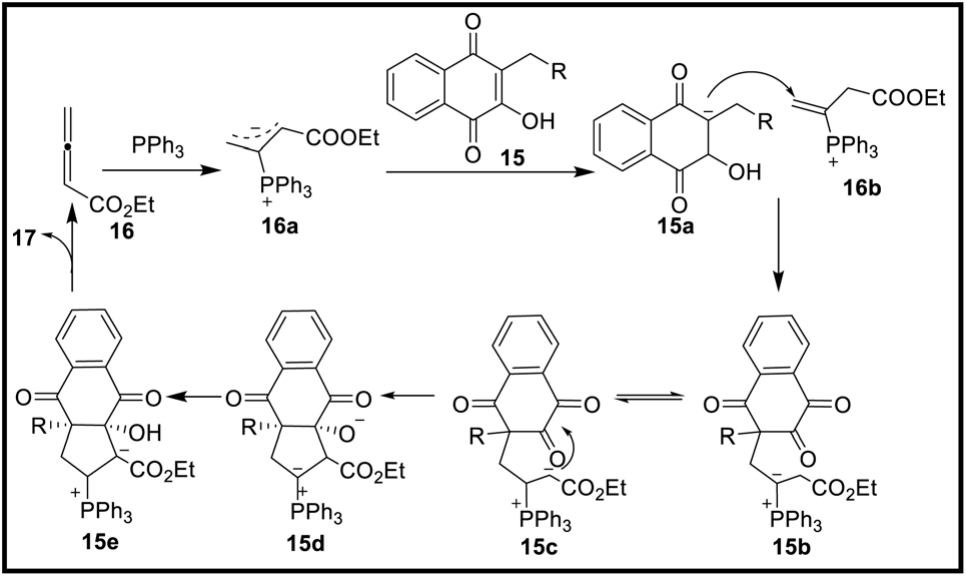
Mechanistic insight into cascade [3 + 2] annulation reaction of allenoate with hydroxynaphthaquinone.

A mechanistic rationale for the formation of [3 + 2] annulation products 17 was proposed (Scheme 6), revealing an unconventional pathway. Nucleophilic activation generates zwitterion 16a, which deprotonates 15 to form the anionic intermediate 15a; this species undergoes γ-addition to intermediate 16b, followed by proton transfer to furnish 15b. Subsequent intramolecular aldol cyclization and β-elimination of the phosphine catalyst deliver product 17. Steric interactions imposed by the benzyl substituent direct both the γ-addition and aldol cyclization to occur on the same face of the six-membered ring, a key factor underpinning the observed high diastereoselectivity.

**Scheme 7:**
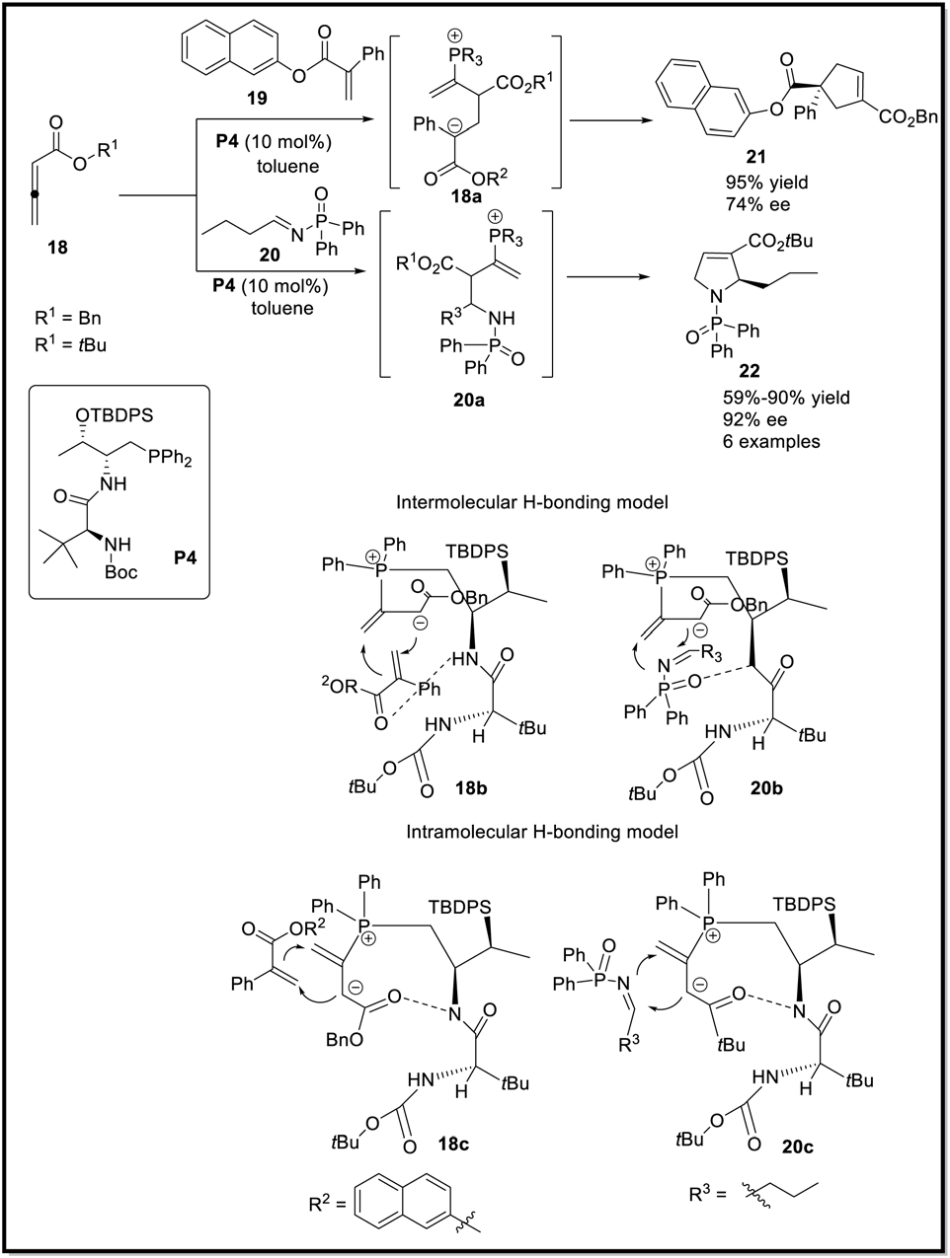
Cascade [3 + 2] annulation reaction of allenoate with acrylate/imine.

In 2018, Yu *et al.* reported a phosphine-catalyzed [3 + 2] annulation of allenoate 18 with acrylates 19 or imines 20, employing a d-Thr-l-*tert*-leu-derived bifunctional phosphine P4 to deliver annulation products 21 and 22 with moderate to excellent enantioselectivity (Scheme 7).^27^ Computational studies reveal that the high stereocontrol arises from precise intra- and intermolecular hydrogen-bonding interactions between P4 and the zwitterionic intermediates 18a and 20a, which govern both regio- and enantioselectivity. In the key step, intramolecular hydrogen bonding, reinforced by non-covalent interactions, directs formation of annulation product 21, whereas intermolecular hydrogen bonding facilitates selective engagement of the amine moiety to afford pyrrole carboxylate derivative 22. This bifunctional activation highlights the role of cooperative non-covalent interactions in orchestrating enantioselective [3 + 2] annulation processes.

**Scheme 8:**
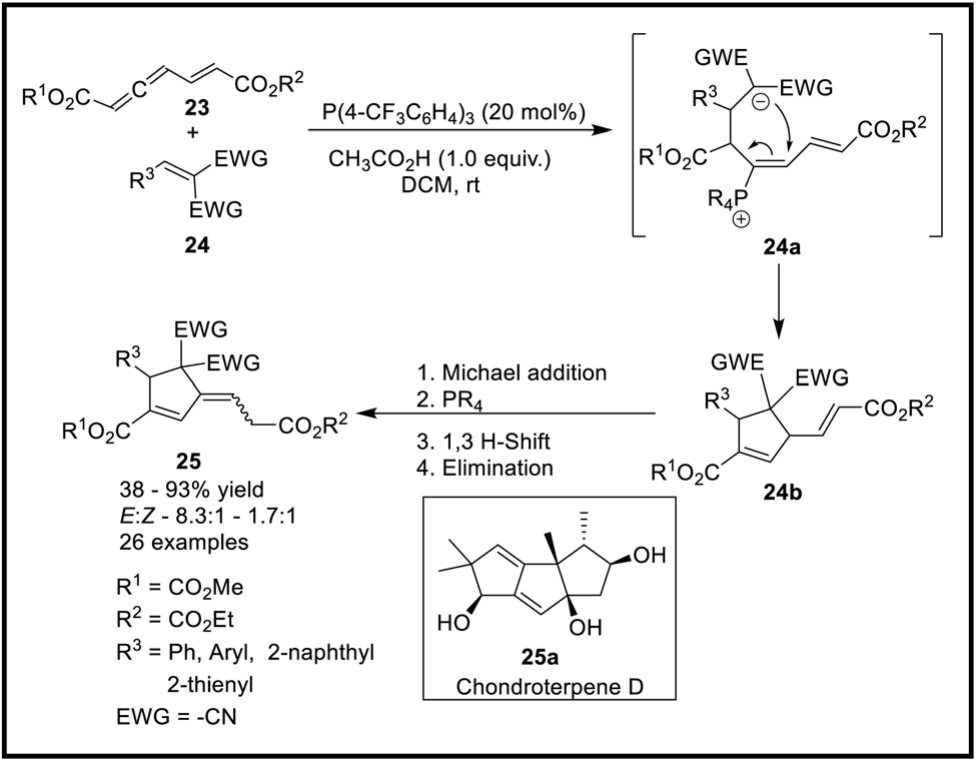
Cascade [3 + 2] annulation of trienedioates with electron-deficient alkenes.

More recently, Li *et al.* reported a one-step phosphine-catalyzed [3 + 2] annulation to construct exocyclic olefinic cyclopentenes 25 from trienedioates 23 and electron-deficient alkenes 24 under mild conditions (Scheme 8).^23^ The transformation proceeds through nucleophilic phosphine addition to allenoate 23, followed by conjugate addition to alkene 24 to form intermediate 24a; subsequent cyclization, a 1,2-hydrogen shift, and phosphine elimination afford pentene intermediate 24b, which undergoes a further phosphine-mediated 1,3-hydrogen shift and elimination to furnish the exocyclic cyclopentene 25. The reaction exhibits moderate *E* : *Z* selectivity, and the resulting scaffold closely resembles the natural product chondroterpene D (25a), underscoring the method's potential for rapid assembly of bioactive cyclopentene frameworks.

**Scheme 9:**
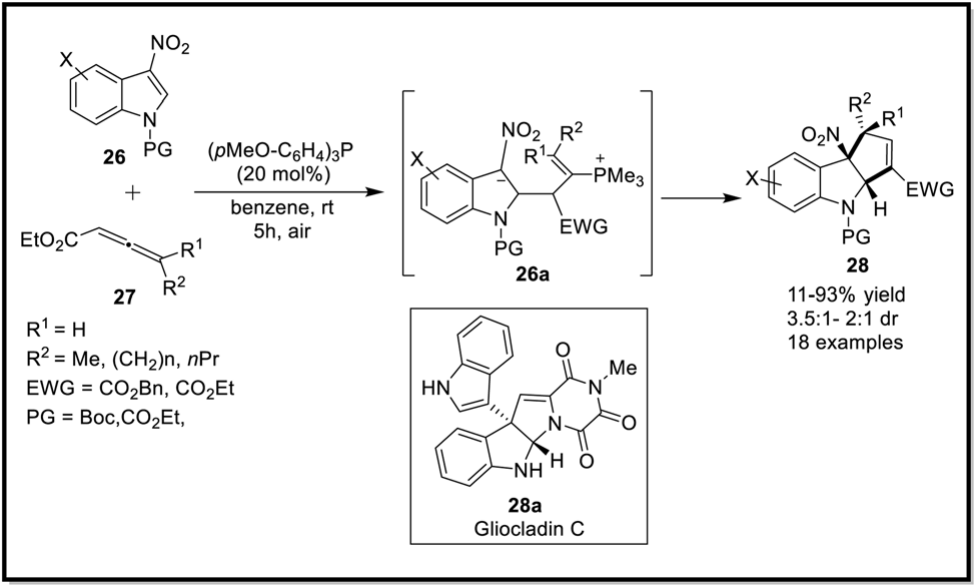
Cascade [3 + 2] annulation of 3-nitroindoles with allenoates.

During this period, several studies explored the reactivity of heterocycles with allenoates to construct fused heterocyclic frameworks. In 2019, Cerveri *et al.* developed a phosphine-catalyzed stereoselective [3 + 2] annulation of 3-nitroindoles 26 with allenoates 27, affording polycyclic fused indoline derivatives 28 in high yields with moderate to good stereochemical control (Scheme 9).^28^ This method enables catalytic dearomatization of indole derivatives, with allenoates bearing electron-withdrawing substituents providing moderate to excellent yields and diastereoselectivities up to 3.5 : 1; electron-donating and electron-withdrawing groups on the indole were well tolerated. Mechanistically, nucleophilic phosphine activation of allenoate 27 generates a zwitterionic intermediate that adds to indole 26 to form intermediate 26a, which undergoes intramolecular dearomative cyclization, water-assisted hydrogen shift, and phosphine elimination to deliver product 28. Computational studies reveal a substantial energy barrier between the *E* and *Z* allenoate intermediates, suppressing *E*–*Z* isomerization and thereby governing the observed diastereoselectivity. Steric effects further reinforce this selectivity. The resulting C2, C3-fused indoline motif is a core structural element in numerous natural products and pharmaceuticals, and notably, 28 closely resembles gliocladin C (28a), a bioactive scaffold with antibacterial, antinematicidal, and anticancer activities. This work could further be extended to the synthesis of fused indoline-based molecular architectures with potential biological relevance.

**Scheme 10:**
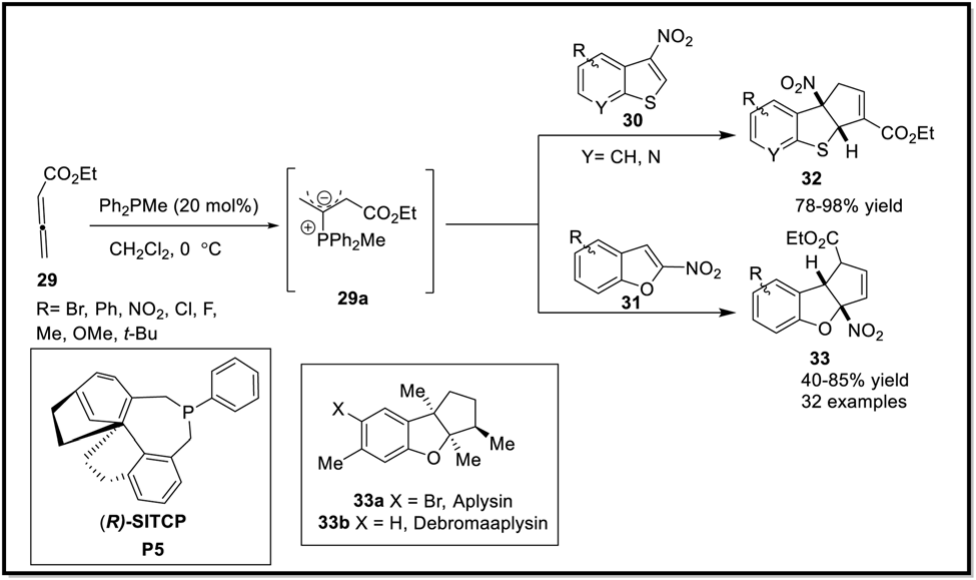
Cascade[3 + 2] annulation of 2-nitrobenzofurans, and 3-nitrobenzothiophenes with allenoates.

Zhao *et al.* further demonstrated that the phosphine-catalyzed dearomative [3 + 2] annulation could be extended to 2-nitrobenzofurans 31 and 3-nitrobenzothiophenes 30, employing Ph_2_PMe as the catalyst to furnish structurally diverse cyclopentabenzofurans 33 and cyclopentabenzothiophenes 32 under mild conditions (Scheme 10).^29^ The study also showcased a chiral phosphine catalyst mediated variant using the chiral phosphine (*R*)-SITCP P5, delivering the product in 63% yield with 11% ee. Mechanistically, nucleophilic addition of the phosphine to the allenoate generates zwitterionic intermediate 29a, which undergoes conjugate addition with 30 or 31, followed by intramolecular cyclization, a 1,2-proton shift, and phosphine elimination to furnish 32 and 33. On appropriate modulation of phosphonium ylide precursors, this strategy could further be extended to the synthesis of cyclopentabenzofuran-based marine sesquiterpene natural products such as aplysin (33a) and debromaplysin (33b).

**Scheme 11:**
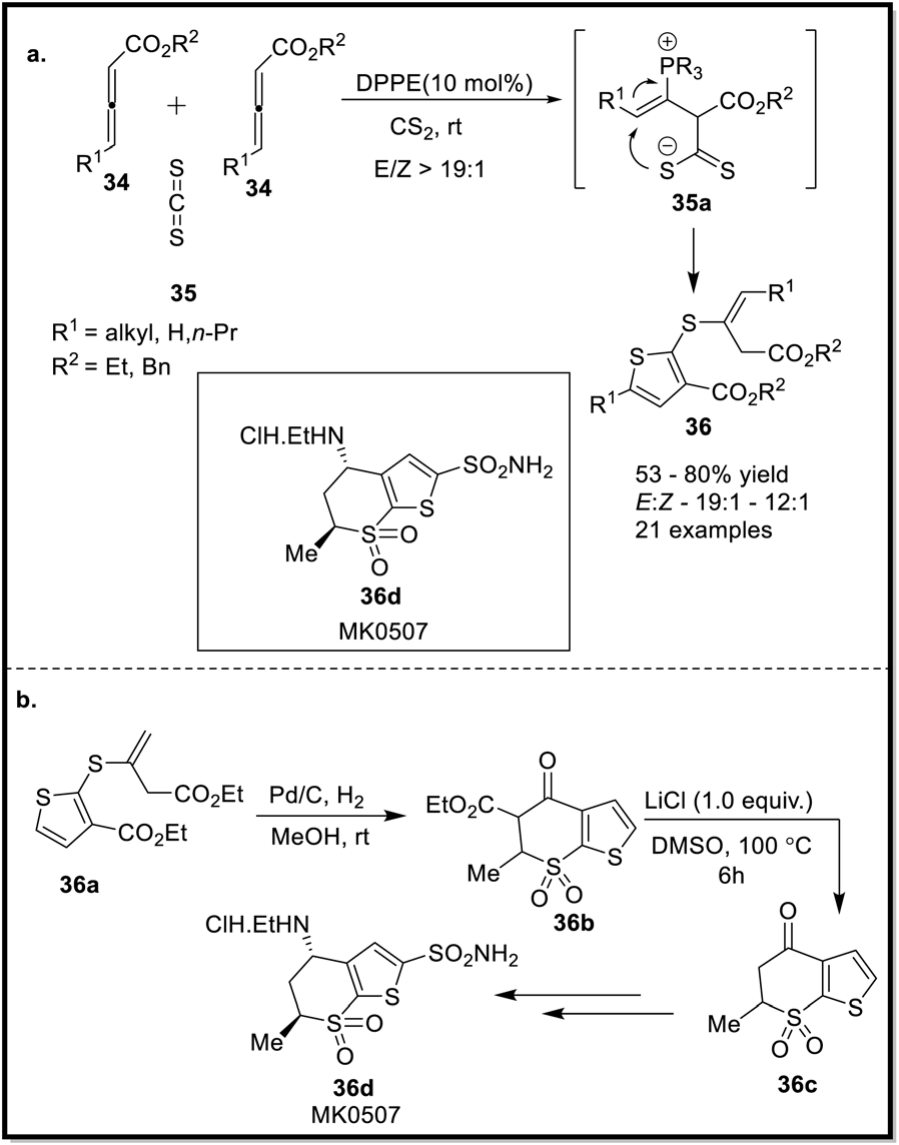
[3 + 2] annulation reaction of γ-substituted allenoate with CS_2_.

Fei and co-workers reported a DPPE-catalyzed three-component [3 + 2] tandem annulation of γ-substituted allenoates 34 with CS_2_35, enabling the efficient synthesis of 2-thienylvinyl sulfides 36 (Scheme 11a).^30^ Both γ-alkyl and γ-aryl allenoates were well tolerated under the reaction conditions. Mechanistically, nucleophilic phosphine activation of allenoate 34 generates a zwitterionic intermediate that reacts with CS_2_ to form intermediate 35a, which undergoes intramolecular Michael addition, 1,2-proton shift, and a second Michael addition with another molecule of 34, ultimately affording product 36 and regenerating the catalyst. This cascade annulation strategy demonstrates synthetic utility for pharmaceutical applications, exemplified by the construction of the antiglaucoma agent MK0507 (36d) *via* cyclization of 2-thienylvinyl sulfide 36a, a key intermediate in the synthesis of 36d (Scheme 11b).

**Scheme 12:**
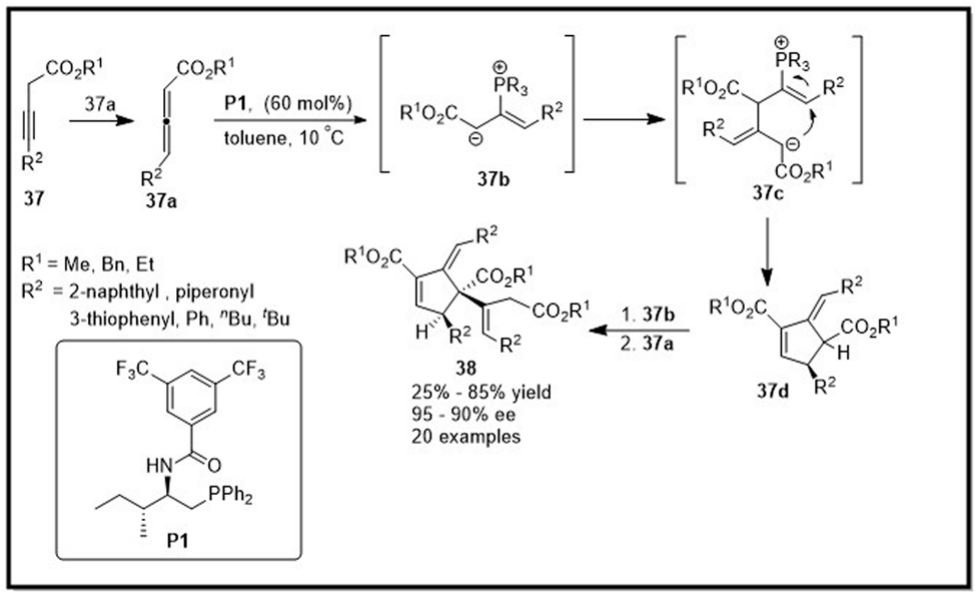
Phosphine-catalyzed trimerization of γ-aryl-3-butynoates.

In 2021, the same group reported an l-isoleucine-derived amide phosphine-catalyzed trimerization of γ-aryl-3-butynoates 37, which undergoes *in situ* isomerization to allenoates 37a, followed by [3 + 2] cyclization and Michael addition to furnish exocyclic cyclopentene derivatives 38 with excellent enantioselectivity and stereospecificity (Scheme 12).^31^ A broad range of γ-aryl-3-alkynoates, encompassing both electron-withdrawing and electron-donating substituents, were compatible, although *ortho*-substituted analogues exhibited reduced reactivity due to steric hindrance. Mechanistically, phosphine activation of allenoate 37a generates zwitterionic intermediate 37b, which serves a dual role as a Brønsted base to isomerize alkynoate 37 and as a nucleophile in the cyclization sequence. Subsequent reaction of 37b with another molecule of 37a forms intermediate 37c, which undergoes intramolecular cyclization, 1,2-proton shift, and catalyst elimination to yield cycloadduct 37d; deprotonation by 37b followed by a final Michael addition with 37a furnishes the exocyclic cyclopentene product 38.

**Scheme 13:**
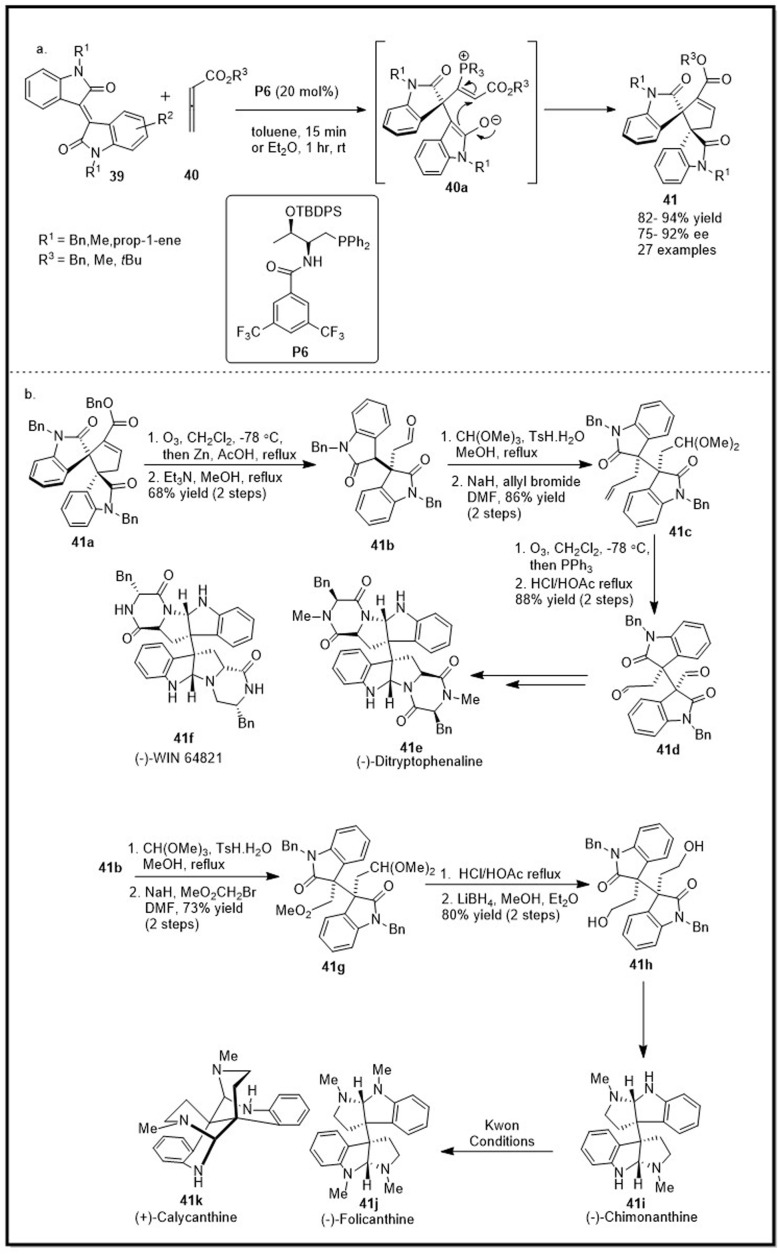
Cascade [3 + 2] annulation of allenes with isoindigos.

Chan and co-workers reported a pioneering phosphine-catalyzed [3 + 2] annulation for the stereoselective construction of spirocyclic bisindoline scaffolds 41 from allenes 40 and isoindigos 39, featuring identical or distinct oxindole moieties (Scheme 13).^32^ The reaction proceeds through a nucleophilic phosphine attack on allene 40, generating a zwitterionic intermediate that undergoes γ-selective addition to 39 to afford intermediate 40a; subsequent intramolecular cyclization and phosphine elimination furnish the spirocyclic bisindoline 41, establishing a contiguous all-carbon quaternary center with high yield and excellent enantiomeric excess. Substrate scope studies revealed that *N*-Ts-protected isoindigos were incompatible with the protocol. The synthetic utility of this strategy was further exemplified in the preparation of cyclotryptamine alkaloids (Scheme 13b); ozonolysis followed by decarboxylation of 41a generates bisoxindole scaffold 41b, which upon acetal formation and allylation yields 41c; subsequent ozonolysis and acetal deprotection afford bisoxindole aldehyde 41d, a versatile intermediate for the synthesis of (−)-ditryptophenaline (41e) and (−)-WIN 64821 (41f). Moreover, acetalation and alkylation of 41b produce 41g, which upon acetal cleavage and reduction delivers bisoxindoline diol 41h, a precursor to (−)-chimonanthine (41i), which can be further elaborated into (+)-calicanthine and (−)-folicanthine. This sequence underscores the broad applicability of phosphine-catalyzed [3 + 2] annulation in stereoselective synthesis of complex natural product frameworks.

**Scheme 14:**
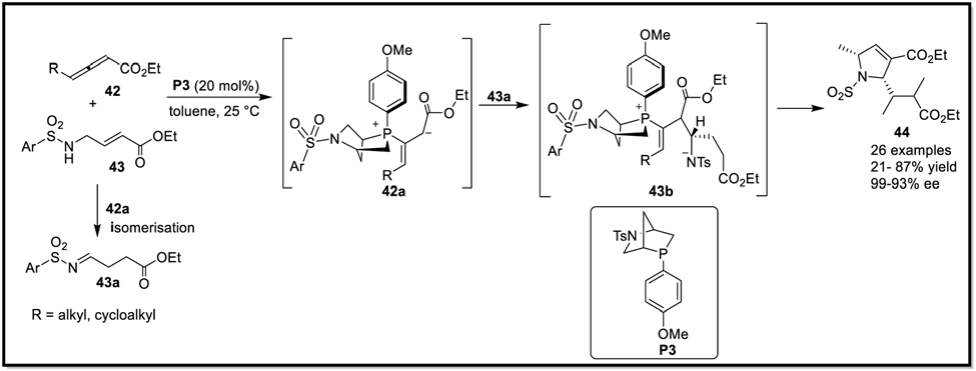
Cascade [3 + 2] annulation reaction of allylamine with allenoate.

In 2021, Zhou and co-workers reported a chiral phosphine-catalyzed [3 + 2] annulation of allylamine 43 with allenoate 42, enabling the stereoselective synthesis of chiral pyrroline derivatives 44 (Scheme 14).^33^ The protocol tolerates a broad range of γ-allenoates, with branched γ-alkyl substituents enhancing reactivity, and proceeds through a tandem isomerization–annulation sequence, providing an efficient and atom-economical route. Mechanistically, nucleophilic phosphine attack on 42 generates zwitterionic intermediate 42a, which deprotonates allylamine 43 to initiate a 1,4-hydrogen shift, yielding species 43a; subsequent *re*-face attack of 42a at the C–N bond forms intermediate 43b, which undergoes intramolecular annulation, a 1,2-proton shift, and phosphine elimination to furnish the chiral pyrroline product 44. DFT calculations revealed that amine to imine isomerization is a prerequisite for productive reaction progression. Furthermore, the observed regioselectivity arises from a synergistic interplay of Lewis acid activation, van der Waals interactions, and steric repulsion, with the favored pathway also being energetically preferred.

**Scheme 15:**
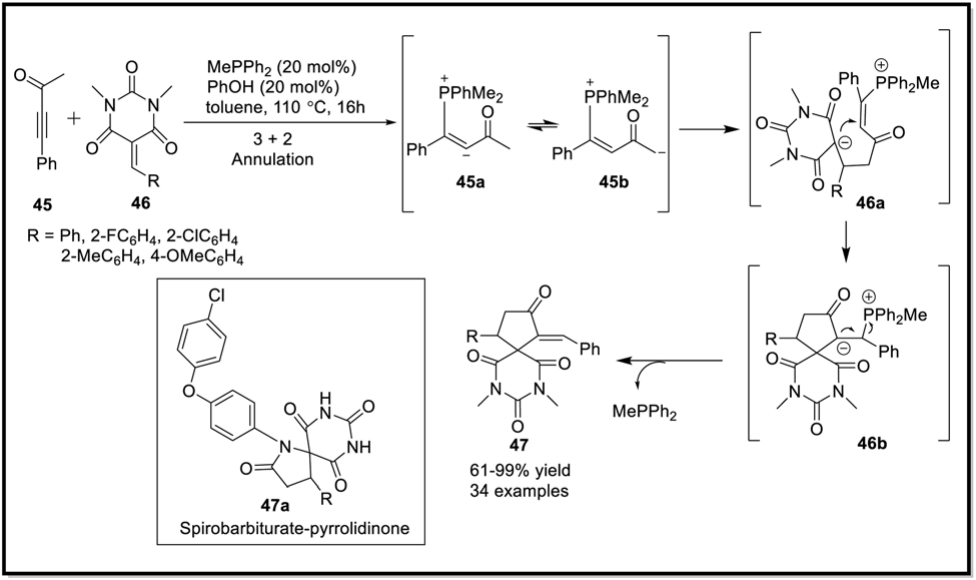
Cascade [3 + 2] annulation of ynones with barbiturate-derived alkene.

Ynones, prevalent motifs in natural products, serve as versatile C2 or C3 synthons in nucleophilic phosphine catalysis. In 2017, Gao and co-workers reported the [3 + 2] annulation of ynones 45 with barbiturate-derived alkenes 46 to construct functionalized spirobarbiturates 47 using methyl diphenylphosphine as the catalyst and phenol as an additive (Scheme 15).^34^ Substrate scope studies revealed that the aromatic substituents on ynones had minimal effect on reactivity, while a wide range of barbiturate-derived alkenes, including naphthyl and heteroaromatic analogues, afforded excellent yields. Mechanistically, nucleophilic attack of the phosphine on 45 generates ylide intermediate 45a, which undergoes a proton transfer to form 45b; conjugate addition of alkene 46 produces 46a, which undergoes intramolecular nucleophilic addition, a 1,2-proton shift facilitated by phenol, and catalyst elimination to deliver spirobarbiturate 47. Notably, base choice can divert the pathway to a [4 + 2] annulation, yielding biologically active pyrano[2,3-*d*]pyrimidine (102, discussed in Section 5, Scheme 34). Notably, the annulation product contains the key spirobarbiturate–pyrrolidinone scaffold present in the bioactive derivatives 47a, which exhibit potent α-glucosidase inhibitory activity. Consequently, these annulation products can serve as valuable synthetic intermediates for the preparation of such biologically active molecules, thereby underscoring both the synthetic versatility and potential medicinal relevance of this annulation strategy.

**Scheme 16:**
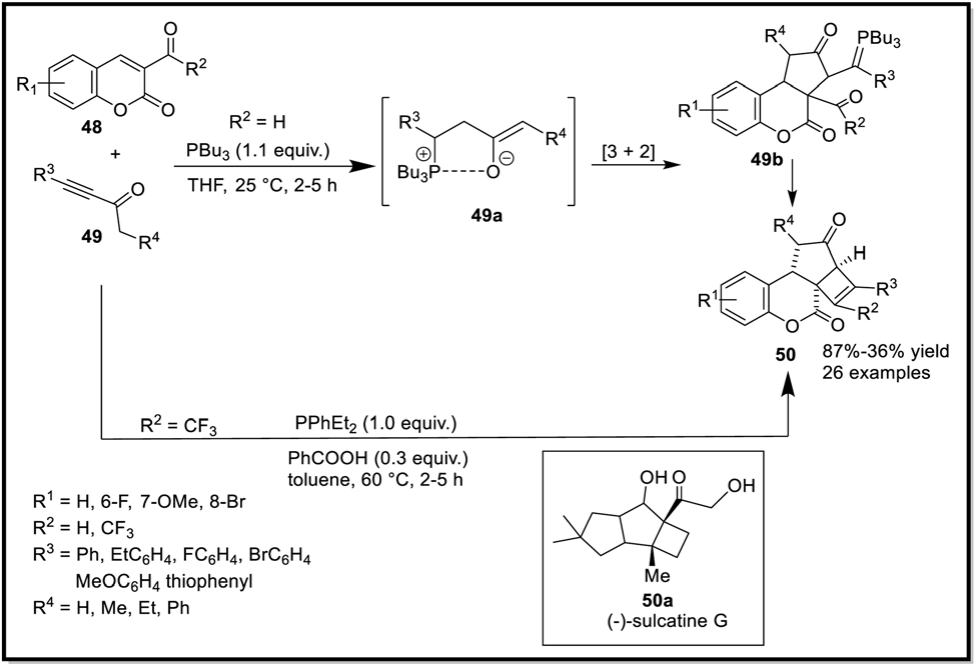
Cascade [3 + 2] annulation of 3-aroylcoumarin with alkynone.

Fu *et al.* reported a phosphine-promoted [3 + 2] annulation strategy for the construction of coumarin-fused bicyclo[3.2.0]heptenones **50**, employing 3-aroylcoumarins 48 and alkynone derivatives 49 (Scheme 16).^35^ This domino intramolecular Wittig [3 + 2] cyclization sequence provides bicyclo[3.2.0]heptenones 50 in moderate to good yields with high regio- and moderate diastereoselectivity (dr = 1.3 : 1). Substrate scope studies demonstrated broad tolerance of coumarin derivatives, with electronic and steric factors exerting minimal influence on reaction efficiency. Moreover, trifluoromethylated aroylcoumarins enabled access to tricyclic scaffolds 50 with high diastereoselectivity upon switching the catalyst from PBu_3_ to PPhEt_2_ in the presence of PhCOOH, irrespective of electronic properties or substitution patterns. Mechanistically, nucleophilic phosphine attack generates ylide intermediate 49a, which undergoes [3 + 2] annulation with 48 to form intermediate 49b, followed by intramolecular Wittig cyclization to furnish product 50. Notably, the resulting tricyclic scaffold closely resembles the natural product sulcatine G (50a), a sesquiterpene isolated from *Laurilia sulcata* with antifungal activity.

**Scheme 17:**
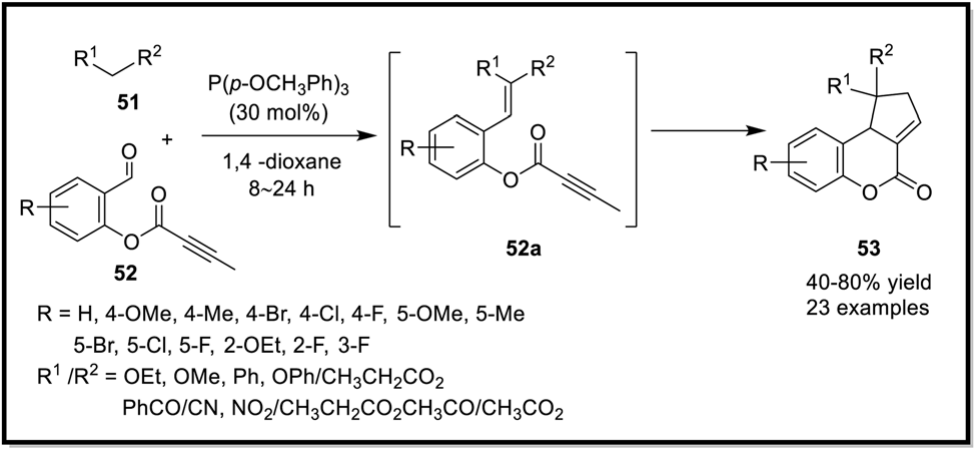
Cascade Knoevenagel condensation through [3 + 2] annulation of 2-formylphenyl alkynoates with activated methylene compounds.

Chen and co-workers developed a phosphine-catalyzed one-pot synthesis of cyclopentene-fused dihydrocoumarins 53 through a cascade classical Knoevenagel condensation through [3 + 2] annulation of 2-formylphenyl alkynoates 52 with activated methylene compounds 51 (Scheme 17).^36^ Alkynoate 52 exhibited a broad range of substrate scope with electron-donating and electron-withdrawing substituents on the phenyl ring. However, the presence of electron-withdrawing substituents at the *ortho*-position lowered the yield. Reactive methylene compound 51 gave a moderate yield regardless of whether it was symmetric or asymmetric. Notably, the asymmetric methylene compounds like ethyl benzoylacetate, benzoylacetonitrile, ethylnitroacetate, and pyrazolone displayed outstanding diastereoselectivity (dr > 20:1), which is attributed to steric hindrance and weak hydrogen bonding between the carbonyl oxygen and the neighboring C–H hydrogen. The reaction proceeds through a trisubstituted styrene intermediate 52a that undergoes nucleophilic phosphine-catalyzed intramolecular [3 + 2] annulation to afford cyclic adduct 53.

**Scheme 18:**
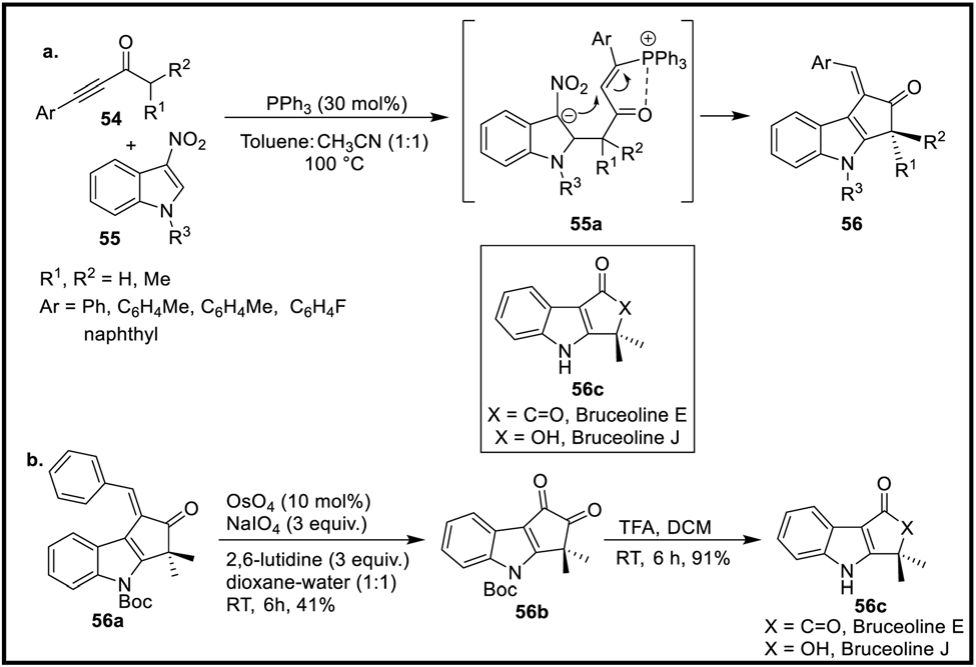
Cascade [3 + 2] annulation of α,β-ynones with 3-nitroindoles.

More recently, Dutta and co-workers reported a metal-free denitrative rearomatizing [3 + 2] annulation of α,β-ynones 54 with 3-nitroindoles 55 to construct α-arylidenecyclopenta[*b*]indoles 56 (Scheme 18).^37^ This transformation enables the formation of tertiary centers as well as sterically encumbered all-carbon quaternary and spirocyclic centers. The methodology was further extended to access 3-benzylidene-cyclopenta[*b*]benzofuran-2-ones, 3-benzylidene-cyclopenta[*b*]-fused benzothiophenes, and α- and β-*N*-indolyl enones, with electron-donating or electron-withdrawing substituents on the ynones only minimally affecting efficiency. Mechanistically, nucleophilic phospha-Michael addition of the phosphine to 54, followed by enolization and addition to 55, generates zwitterionic nitronate intermediate 55a, which undergoes cyclization, a 1,4-hydrogen shift, nitrate elimination, and phosphine elimination to furnish product 56. The strategy was further leveraged for the synthesis of tricyclic natural products bruceolline E and bruceolline J (56c), bioactive scaffolds historically employed in malaria and other parasitic disease treatments; in this sequence, oxidative cleavage of the benzylidene group with OsO_4_/NaIO_4_ followed by *N*-protecting group removal using TFA furnished the final natural product 56c (Scheme 18b). This investigation features a unique work emphasizing the need of asymmetric phosphine organocatalysis in complex natural product synthesis.

**Scheme 19:**
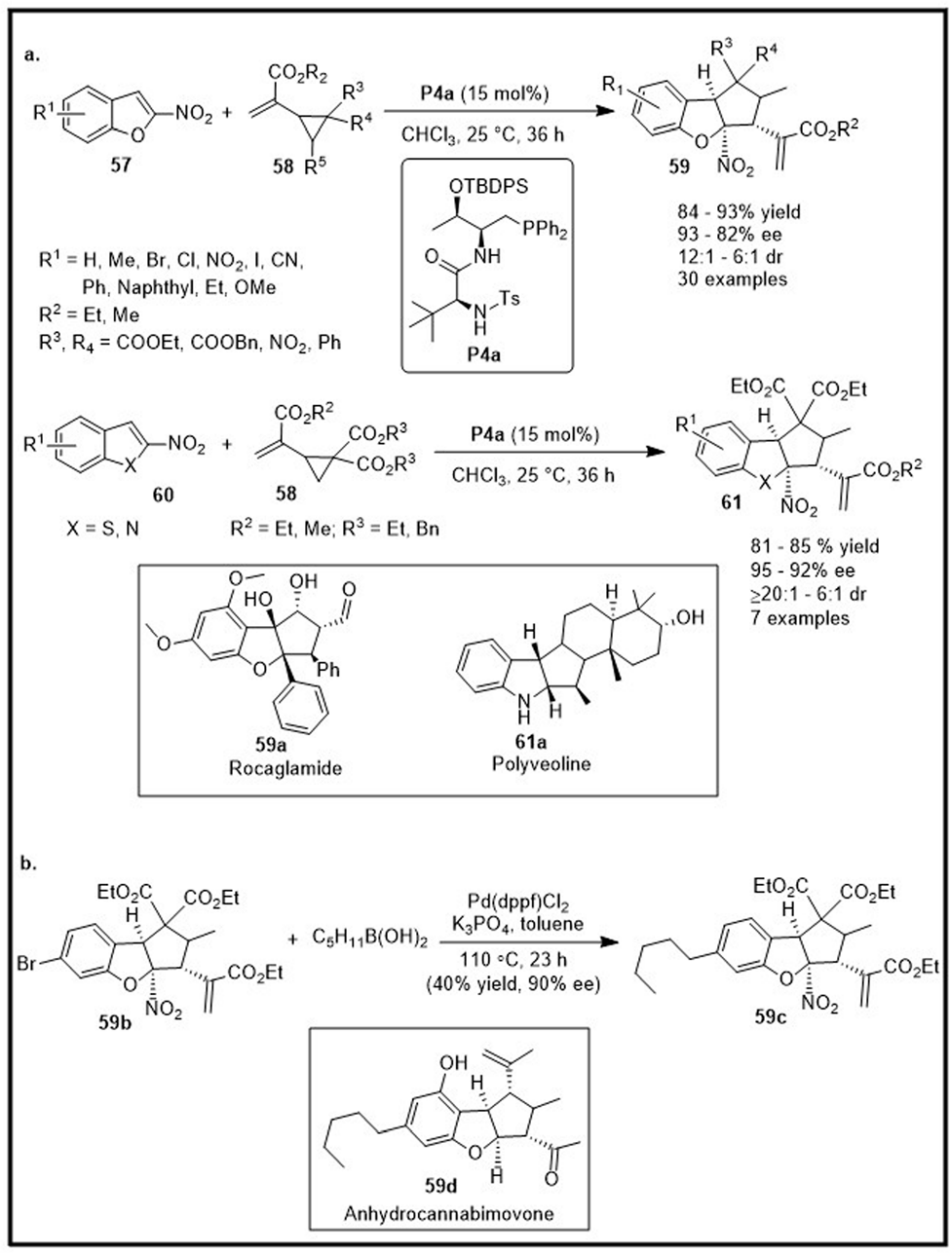
Cascade dearomative [3 + 2] annulation of heteroarenes with vinylcyclopropanes.

Despite extensive exploration of MBH carbonates, allenoates, and alkynones, recent efforts have focused on expanding the repertoire of electrophilic substrates for nucleophilic phosphine catalysis. In 2023, Dai *et al.* reported a phosphine-catalyzed dearomative [3 + 2] annulation of diverse heteroarenes—including benzofurans 57, benzothiophenes, and indoles 60 with vinylcyclopropanes 58 to access chiral cyclopentabenzodihydrofurans 59 and cyclopentaindoline scaffolds 61 with high diastereo- and enantioselectivity (Scheme 19).^37^ Substrate scope studies showed broad tolerance, with nitrobenzofurans bearing electron-donating and electron-withdrawing groups, as well as vinylcyclopropanes with diverse ester substituents, providing consistently high selectivity. 2-Nitrobenzothiophenes and 3-nitroindoles also furnished products in excellent yields. DFT studies revealed that the preferential formation of the major stereoisomer arises from *re*-face attack at the less sterically hindered site. Notably, the resulting scaffolds share structural similarity with natural products such as rocaglamide 59a and polyveoline 61a, the former exhibiting anticancer, anti-inflammatory, and insecticidal activity. The synthetic utility of the protocol was further demonstrated by the construction of an anhydrocannabimovone-core containing product 59d through the Suzuki coupling of 59 (Scheme 19), highlighting the method's potential in assembling complex, bioactive molecular architectures.

**Scheme 20:**
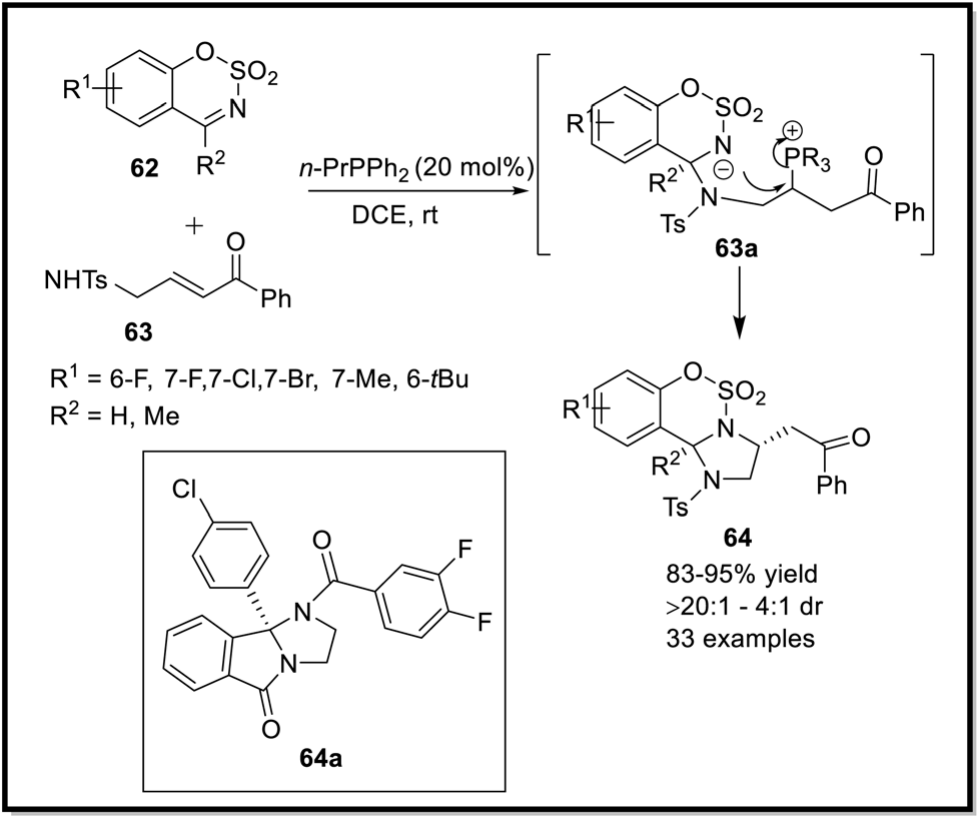
Cascade [3 + 2] annulation of β sulfonamido-substituted enones with sulfamate-derived cyclic imine.

In the same year, Shi and co-workers reported a phosphine-catalyzed [3 + 2] annulation for the synthesis of imidazoline derivatives 64 from β-sulfonamido-substituted enones 63 and sulfamate-derived cyclic imines 62 (Scheme 20).^38,39^ The reaction is initiated by nucleophilic phosphine attack on enone 63 to form a phosphonium intermediate, which undergoes proton transfer and nucleophilic addition to imine 62, generating intermediate 63a; subsequent catalyst regeneration furnishes the imidazoline product 64. The protocol exhibits broad substrate scope, accommodating various substituted enones and cyclic imines, with dichlorosubstituted enones showing high reactivity and halogen-substituted cyclic imines affording excellent yields and diastereoselectivity. The observed stereocontrol arises from steric interactions between the benzene and benzoyl methyl groups, as well as between the sulfonyl and benzoyl methyl groups during the intramolecular cyclization. Notably, the resulting imidazoline 64 displays structural similarity to biologically active derivative 64a, known for its anti-colon cancer and anti-leukemia activity.^40^

## 3. Asymmetric cascade reactions involving [3 + 3]-annulation

Despite their underexplored nature relative to [3 + 2] and [4 + 2] annulations, [3 + 3] cycloadditions have emerged as a powerful platform for six-membered heterocycle construction, exemplified by the report of Zheng *et al.* in 2009 on phosphine-catalyzed transformation of *tert*-butyl allylic carbonates with alkylidenemalononitriles. This work marked a milestone that has seeded asymmetric variants and enriched the collective corpus of organophosphine-catalyzed cycloaddition reactions. In [3 + 3] annulation, two complementary three atom synthons combine together to generate a six-membered ring.

**Scheme 21:**
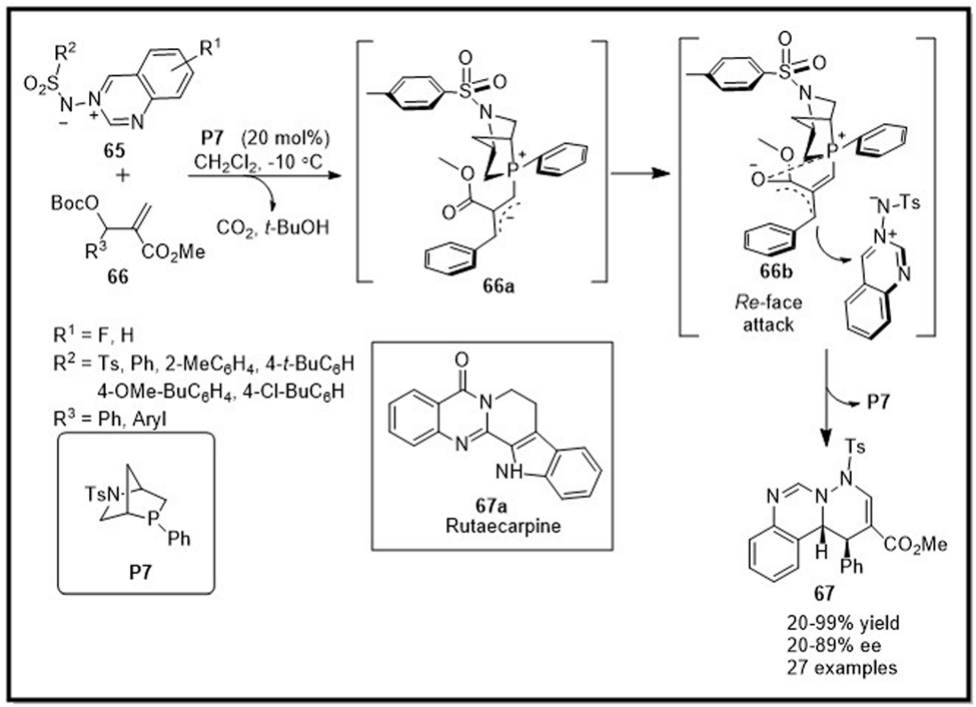
Cascade [3 + 3] annulation of quinazoline-based 1,3-dipoles with MBH carbonates.

In 2017, Zhou and co-workers reported an enantioselective phosphine-catalyzed [3 + 3] annulation of quinazoline-based 1,3-dipoles 65 with MBH carbonates 66 to afford chiral cycloadducts 67 in high yield and excellent enantioselectivity (Scheme 21).^41^ Substrate scope studies revealed broad tolerance of aryl substituents on both MBH carbonates and azomethine imines, with *para*-nitro aryl MBH carbonates showing high reactivity, while alkyl-substituted MBH carbonates remained inert. Sulfonyl protecting groups with *para-tert*-butyl substitution delivered 97% yield and 90% ee. Mechanistically, nucleophilic attack of the phosphine on 66 generates intermediate 66a, which undergoes isomerization followed by reaction with the imine 65, intramolecular Michael addition, and catalyst elimination to furnish 67. The high enantioselectivity arises from *re*-face attack of the imine, reinforced by steric effects imparted by the phosphine catalyst. Notably, the cycloadduct 67 bears structural resemblance to the quinazolino-carboline alkaloid rutaecarpine (67a), a bioactive scaffold exhibiting anti-inflammatory, anticancer, and vasodilatory activity, highlighting the potential of this strategy for constructing quinazoline-based natural products and bioactive molecules.

**Scheme 22:**
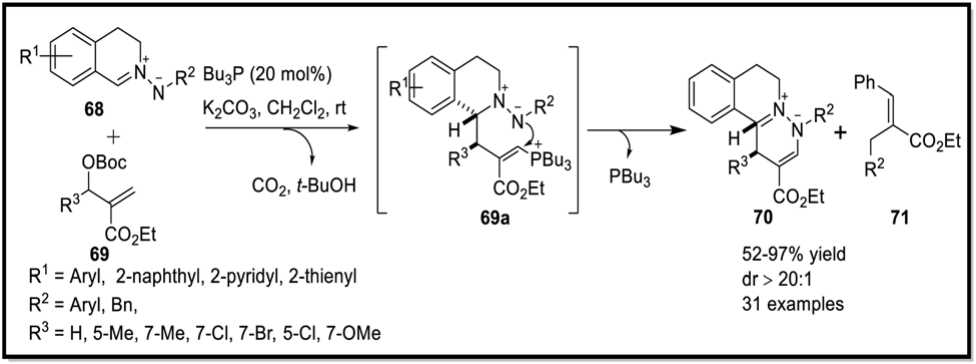
Cascade [3 + 3] annulation of azomethine imine with various MBH carbonates.

The same group further developed a phosphine-catalyzed [3 + 3] annulation of azomethine imine 68 with various MBH carbonates 69 to construct isoquinoline scaffolds 70, with PBu_3_ identified as an efficient catalyst (Scheme 22).^42^ The reaction predominantly yielded cycloadduct 70, alongside trace amounts of addition product 71. Both MBH carbonates 69 and cyclic imines 68 tolerated a broad array of aryl substituents, delivering high yields and good to excellent diastereoselectivity, while alkyl-substituted MBH carbonates remained poorly reactive. Mechanistically, nucleophilic attack of the phosphine on 69 forms ylide intermediate, which reacts with 68 to generate intermediate 69a; subsequent intramolecular conjugate addition and phosphine elimination afford the isoquinoline 70. The observed *trans*-diastereoselectivity arises from steric hindrance between the aryl substituents and the tetrahydroisoquinoline framework during ylide formation. Additionally, a pronounced base effect was noted, presumably facilitating the generation of the allylic ylide species, highlighting the nuanced interplay between catalyst, substrate, and base in achieving selective [3 + 3] annulation.

**Scheme 23:**
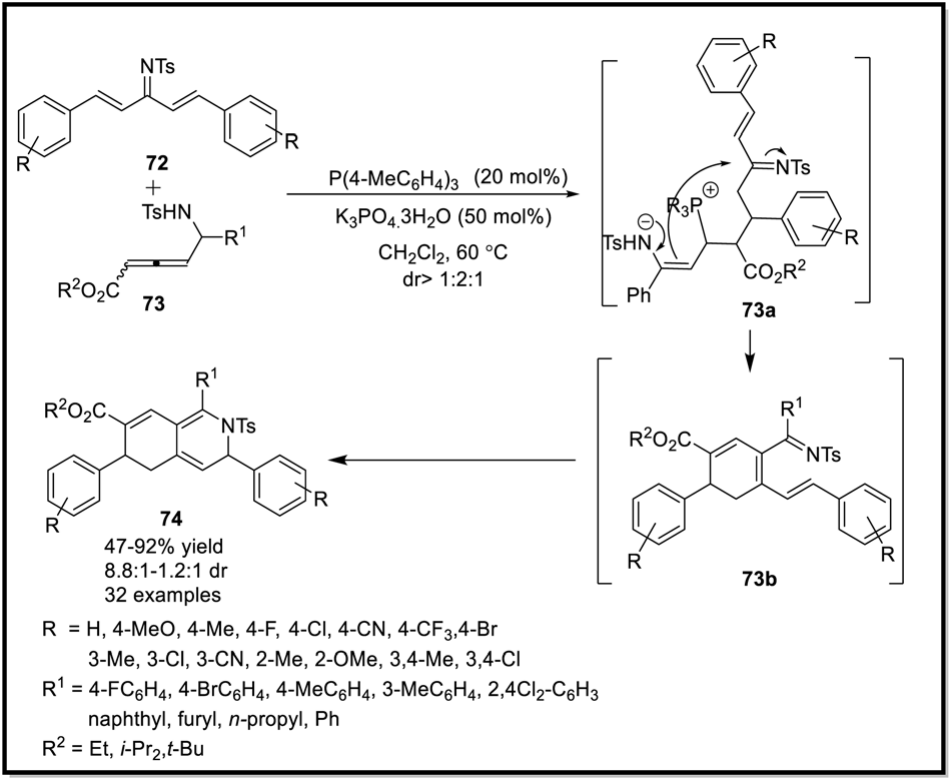
Cascade [3 + 3] annulation of cross-conjugated azatrienes with δ-sulfonamido allenoate.

In 2020, Li *et al.* reported a pioneering tandem [3 + 3] annulation/aza-6π-electrocyclization of cross-conjugated azatrienes 72 with δ-sulfonamido allenoates 73, affording functionalized isoquinoline derivatives 74 in good yields and diastereoselectivity (Scheme 23).^43^ The protocol demonstrated broad substrate tolerance, accommodating azatrienes with electron-donating or electron-withdrawing aryl groups, aliphatic substituents, and sulfonamido derivatives. Mechanistically, nucleophilic phosphine attack at the β-position of 73 initiates the reaction, followed by proton shifts, isomerization, and Michael addition to 72; subsequent [3 + 3] annulation, proton transfer, and phosphine elimination furnish intermediate 73b, which undergoes an aza-6π-electrocyclization to generate isoquinoline 74. This elegant sequence highlights the potential of organophosphine mediated tandem annulation/electrocyclization strategies for the efficient construction of diversely functionalized isoquinoline scaffolds. The [3 + 3] annulation strategy has emerged as a powerful platform for constructing bioactive N-heterocycles, particularly isoquinoline scaffolds, and while imines have dominated as C3 synthons, the exploration of alternative electrophilic partners holds considerable promise to further expand the synthetic repertoire and enrich the collective scientific corpus.

## 4. Asymmetric cascade reactions involving [4 + 1]-annulation

Phosphine-catalyzed [4 + 1] annulations have emerged as a compelling complement to [3 + 2] strategies for constructing five-membered carbo- and heterocycles, offering access to molecular architectures that are challenging to achieve through conventional [3 + 2] routes.^44^ Despite the well-established nature of [3 + 2] annulations, reports on [4 + 1] transformations, where a four atom synthon combines with a single atom synthon, remain comparatively scarce, underscoring the untapped potential of this approach. Mechanistically, these reactions involve phosphine-promoted coupling of C1 synthons with four-atom conjugated systems, with common substrates including electron-deficient allenes and modified MBH adducts. Allenoates exhibit dual reactivity as either C1 or C4 synthons, whereas maleimides and 1,2-dicarbonyl compounds predominantly act as C1 partners, underscoring the versatile and modular character of [4 + 1] annulations and their potential to broaden the landscape of organophosphine mediated cyclizations.

**Scheme 24:**
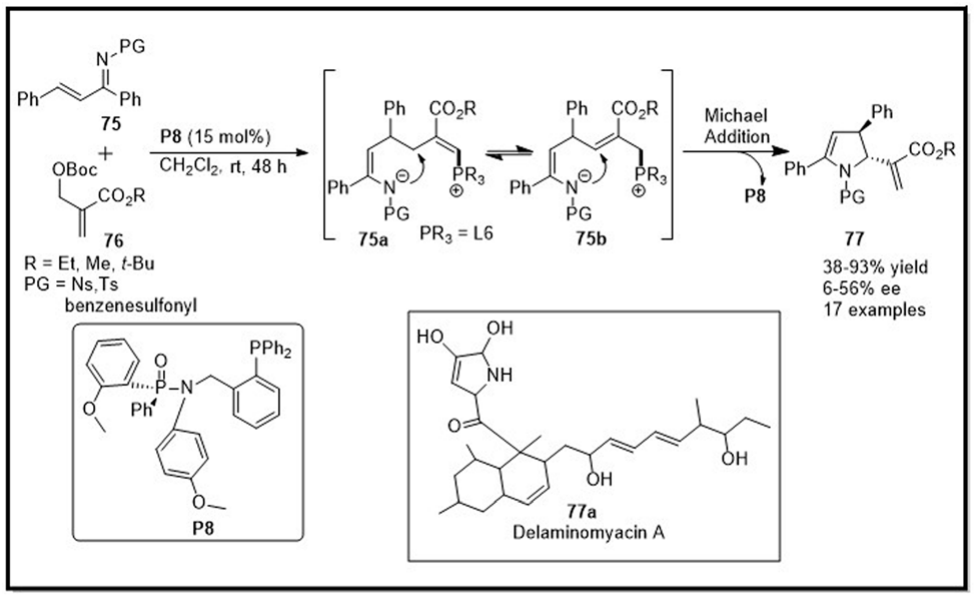
Cascade [4 + 1] annulation reaction of α,β-unsaturated imines with allylic carbonates.

In 2017, Li and co-workers reported an asymmetric [4 + 1] annulation of α,β-unsaturated imines 75 with allylic carbonates 76 using a novel hybrid P-chiral oxidephosphine catalyst P8 to furnish polysubstituted 2-pyrroline derivatives 77 in good yields and high enantioselectivity (Scheme 24).^45^ The reaction accommodates a broad array of aryl-substituted imines, with stereoselective γ-carbanion addition of the ylide to 75 generating intermediate 75a, which interconverts to 75b; subsequent intramolecular Michael addition and catalyst elimination delivers 77. The good enantioselectivity is attributed to stereocontrolled conjugate addition and intramolecular coulombic interactions between the phosphonium unit and the chiral phosphine moiety of P8. Notably, the cycloadduct 77 bears structural resemblance to the immunomodulatory and antibacterial natural product delaminomycin A. This work further affirms the capacity of small-molecule organophosphine catalyst in constructing architecturally alluring bioactive molecular frameworks.

**Scheme 25:**
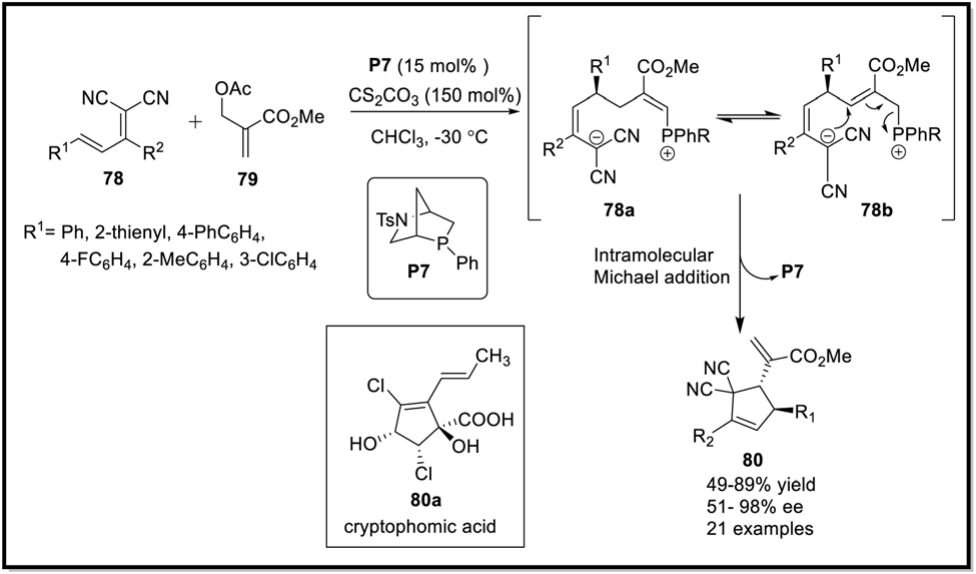
Cascade [4 + 1] annulation of polar diene with allylic derivatives.

In 2021, the same group extended the asymmetric [4 + 1] annulation strategy by employing polar dienes 78 with allylic derivatives 79 under the promotion of cyclic phosphine P7 to construct enantioenriched carbocyclic adducts 80 (Scheme 25).^46^ A broad range of 1,3-dienes was well tolerated, delivering moderate to good yields and moderate to excellent enantioselectivities, with conjugated substituents such as aryl groups at the 2-position proving essential, whereas methyl- or hydrogen-substituted dienes showed poor reactivity. Mechanistically, γ-carbanion addition of the *in situ* generated ylide to 78 forms intermediate 78a, which tautomerizes to 78b; subsequent cyclization and phosphine elimination furnish 80, with preferential *si*-face addition dictating the observed enantioselectivity. The cyclopentene adduct 80 bears structural similarity to the marine natural product cryptophomic acid 80a, an antimicrobial agent. This protocol further demonstrates that appropriate tuning of polar dienes in combination with the P7 catalyst provides an efficient and stereocontrolled route to cyclopentene-based natural products.

**Scheme 26:**
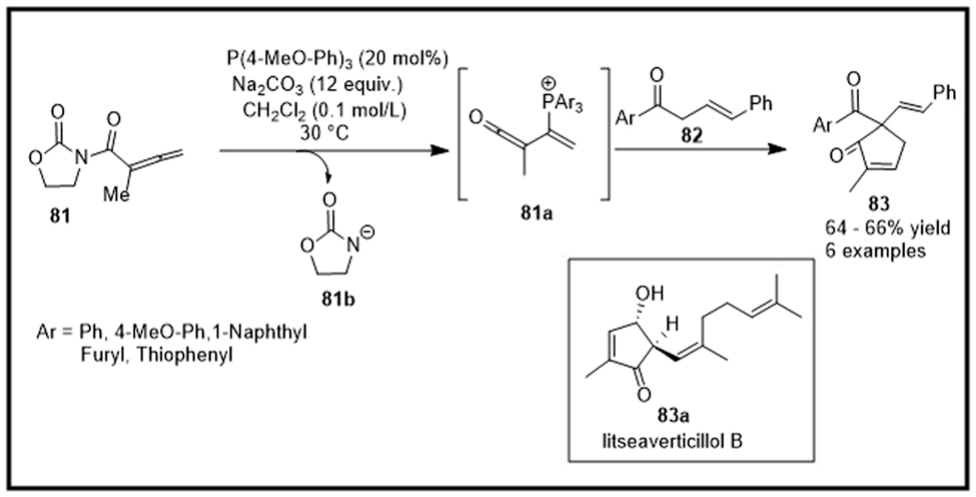
Cascade [4 + 1] annulation of allenyl imide with β,γ-enone.

More recently, Xiang and co-workers reported a phosphine-catalyzed chemodivergent [4 + 1] annulation of allenyl imide 81 with β,γ-enone 82 to furnish 2-cyclopentenone derivatives 83 in moderate yields (Scheme 26).^47^ P(4-MeO-Ph)_3_ efficiently catalyzed the transformation, tolerating ketones bearing electron-withdrawing aryl groups and heteroaryl substituents. Mechanistically, nucleophilic attack of the phosphine on 81, followed by elimination of the 2-oxazolidinyl anion 81b, generates ketenyl vinyl phosphonium species 81a; deprotonation of 82 by 81b or base renders it nucleophilic, enabling attack on 81a. Sequential 1,3-proton transfer, intramolecular Michael addition, and isomerization, coupled with catalyst regeneration, afford 83. Notably, switching to an amine catalyst in the presence of DABCO and Cs_2_CO_3_ triggers chemodivergence, producing [3 + 3] pyranose scaffolds instead of cyclopentenones. The cyclopentenone 83 resembles the anti-HIV natural product litseaverticillol B 83a. Organophosphine-mediated [4 + 1] annulations thus offer an efficient route to synthesize cyclopentene derivatives, and their extension to diverse diene frameworks presents a promising avenue for accessing unconventional molecular architectures.

## 5. Asymmetric cascade reactions involving [4 + 2]-annulation

Phosphine-catalyzed [4 + 2] annulations, where a four atom synthon combines with a two atom synthon, have emerged as a powerful and convenient strategy for the construction of six-membered hetero- and carbocyclic frameworks. This approach was first reported by Kwon in 2003, who demonstrated the annulation of α-substituted allenoates with *N*-tosylaldimines to afford tetrahydropyridine rings.^48^ In the early development of this methodology, substituted allenoates predominantly served as C4 synthons, while imines functioned as efficient C2 partners.^49,50^

**Scheme 27:**
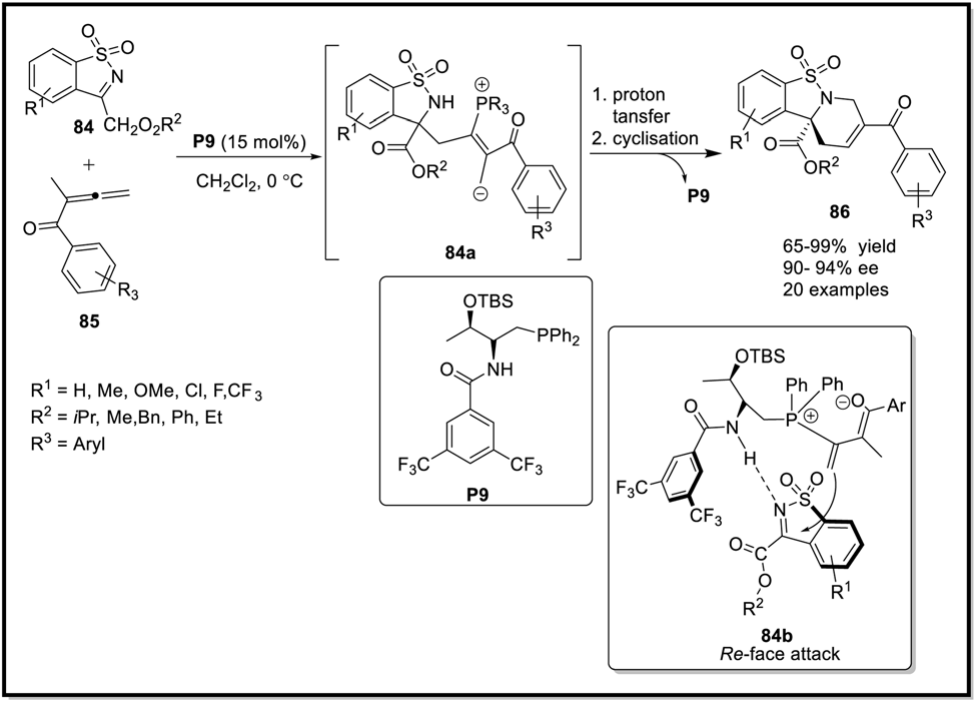
Cascade [4 + 2] cycloaddition of α-substituted allenic ketones with *N*-sulfonyl cyclic ketimines.

In 2019, Wang and co-workers reported an asymmetric [4 + 2] cycloaddition of α-substituted allenic ketones 85 with *N*-sulfonyl cyclic ketimines 84 under the promotion of amino-acid-derived bifunctional phosphine P9 to afford sultam-fused tetrahydropyridines 86 bearing a quaternary stereocenter in good yields and excellent enantioselectivities (Scheme 27).^51^ A broad range of *N*-sulfonyl cyclic ketimines, including aryl ketones and six-membered ring imines, was well tolerated. Notably, the phenyl substituent on the allenic ketone 85 influenced reactivity but not enantioselectivity. Mechanistically, nucleophilic attack of P9 on 85 generates a phosphonium enolate intermediate, which engages 84 to form intermediate 84a. The bifunctional catalyst adopts a conformation conducive to hydrogen bonding with 84, directing a *re*-face attack 84b and governing stereoselectivity. Subsequent proton transfer, intramolecular cyclization, and catalyst regeneration furnish the tetrahydropyridine product 86.

**Scheme 28:**
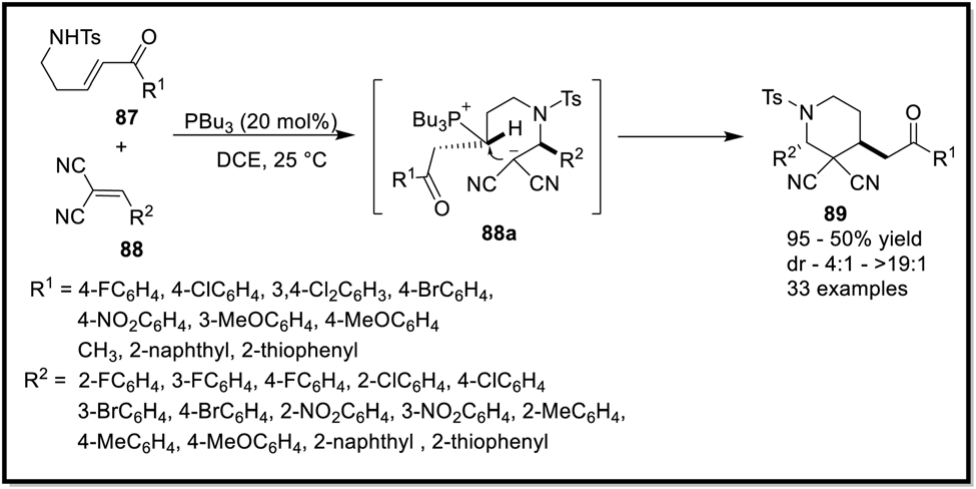
Cascade [4 + 2] annulation of sulfonamido-substituted enones with dicyanoalkene.

In 2021, Liu and co-workers reported a phosphine-catalyzed [4 + 2] annulation between sulfonamido-substituted enones 87 as C4 synthons and electron-deficient dicyanoalkenes 88, affording piperidine derivatives 89 in good to excellent yields with moderate to excellent diastereoselectivity (Scheme 28).^52^ Dicyanoalkenes bearing halogen-substituted aryl groups delivered the highest yields, whereas electron-donating substituents reduced efficiency. Mechanistically, nucleophilic attack of PBu_3_ on 87, followed by proton transfer, generates a zwitterionic intermediate that attacks 88 to give 88a. Subsequent intramolecular nucleophilic cyclization and phosphine elimination furnish the piperidine product 89. Steric interactions between the R^1^ group and the benzoyl methyl moiety in 88a favor the *trans*-isomer as the major product, thereby controlling diastereoselectivity.

**Scheme 29:**
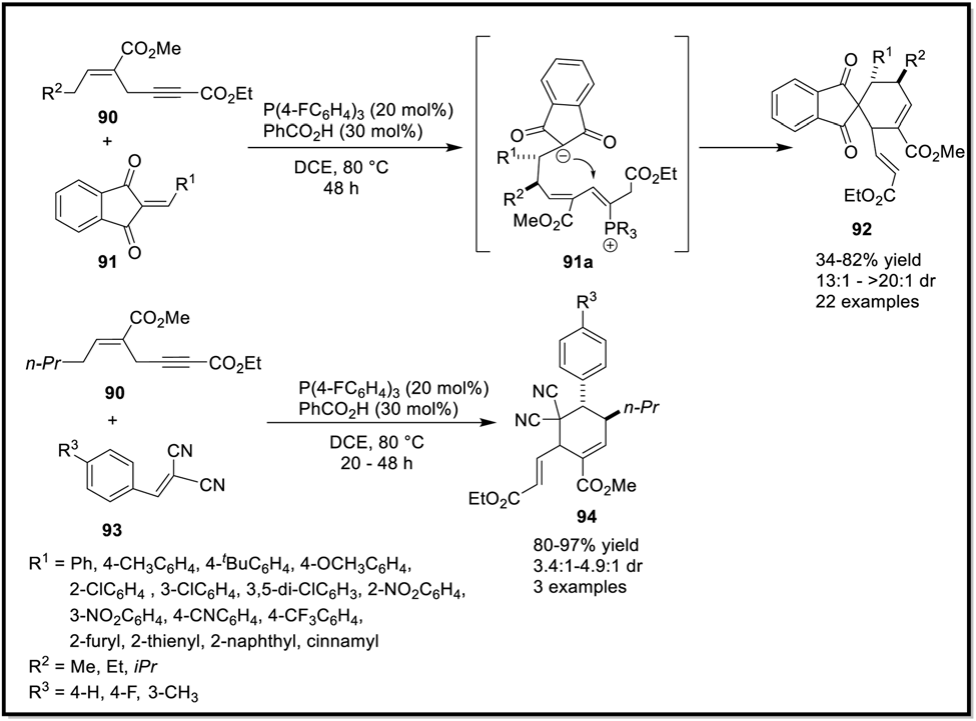
Cascade [4 + 2] annulation of 1,4-enynoates with electron-deficient olefins.

Around the same time, Xu *et al.* reported a phosphine-catalyzed [4 + 2] annulation of 1,4-enynoates 90 with electron-deficient olefins 91 and 93, employing P(4-FC_6_H_4_)_3_ as the catalyst to afford highly functionalized cyclohexene derivatives 92 and 94, respectively, in moderate to good yields with high diastereo- and regioselectivity (Scheme 29).^53^ Notably, the protocol proceeds *via* an internal redox [4 + 2] annulation, wherein γ- and ω-C(sp^3^)−H bonds of the enynoate undergo oxidation, while the acetylenic moiety is concomitantly reduced. Substrate scope studies revealed that (arylmethylidene)indane-1,3-diones tolerate a broad array of aryl and heteroaryl substituents, whereas alkyl-substituted derivatives were unreactive. Remarkably, 1,4-enynoates lacking a δ-ester group preferentially furnished [3 + 2] products, highlighting the critical role of the ester functionality in directing the [4 + 2] reaction pathway.

**Scheme 30:**
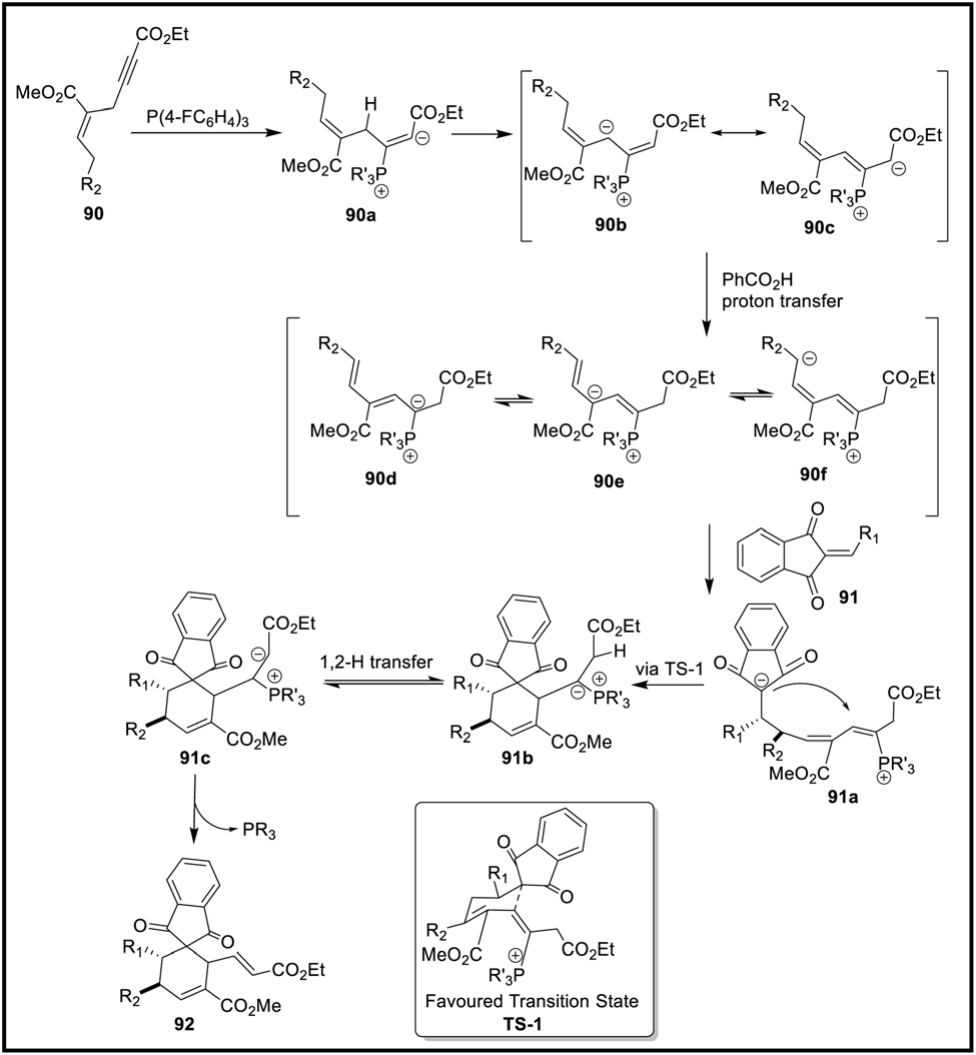
Mechanistic rationale for the [4 + 2] annulation of 1,4-enynoates with electron-deficient olefins.

The mechanistic rationale is depicted in Scheme 30. The reaction is initiated by nucleophilic attack of the phosphine on 90, generating vinyl anion intermediate 90a. Deprotonation at the γ-carbon forms resonance-stabilized zwitterionic intermediates 90b and 90c, which undergo sequential proton transfers to give the ϕ-carbanion intermediate 90f. Subsequent addition of 90f to olefin 93 generates ylide intermediate 91a, which undergoes a 6-*exo-trig* cyclization followed by a 1,2-hydrogen shift and phosphine elimination to afford the cyclohexene product 94. The observed diastereoselectivity arises from steric effects that favor the *exo-trig* cyclization *via* the sterically preferred transition state TS1.

**Scheme 31:**
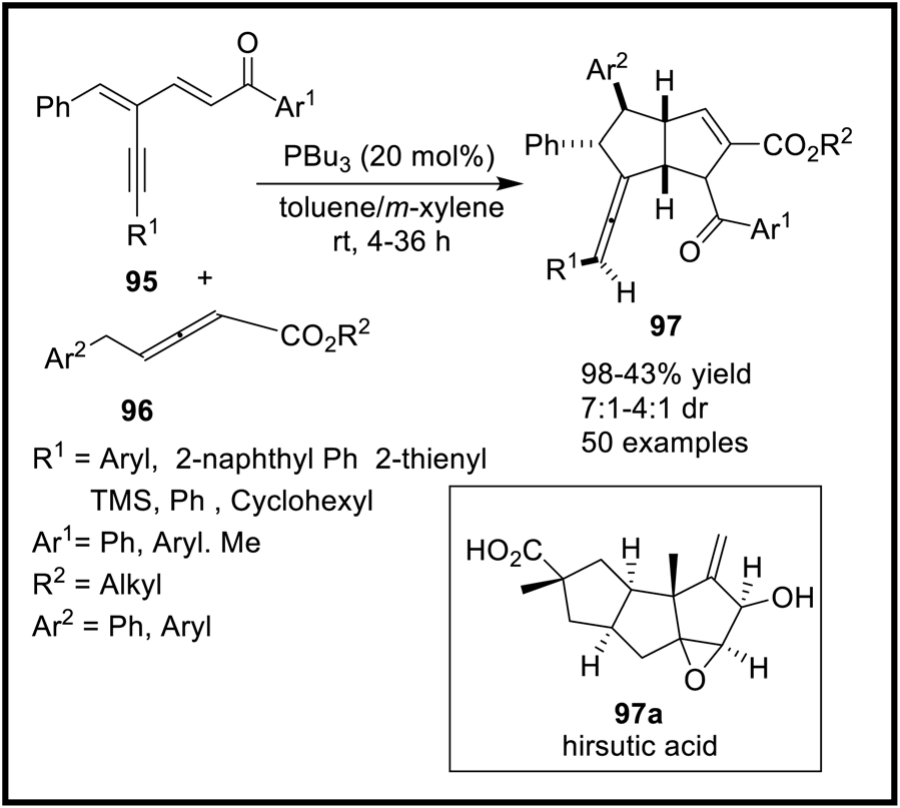
Cascade [4 + 2] annulation of enynes with γ-substituted allenoates.

Building on Xu *et al.*'s demonstration of conjugated enynes as effective C4 synthons, Tang and co-workers in 2023 developed a diastereoselective phosphine-catalyzed [4 + 2] annulation of enynes 95 with γ-substituted allenoates 96, enabling the construction of allenes embedded within hexahydropentalene frameworks 97 (Scheme 31).^54^ The cascade isomerization/annulation protocol tolerated a broad range of aryl-substituted enynes bearing both electron-donating and electron-withdrawing groups; however, alkynyl substituents such as TMS, 2-pyridyl, or alkyl groups led to poor reactivity, likely due to desilylation or Lewis base interference with the allenoate. Notably, γ-alkyl allenoates favored classical [3 + 2] annulation, resulting in distinct regioselectivity. The resulting adduct 97 exhibits structural similarity to hirsutic acid 97a, a natural product with pronounced antitumor activity.

**Scheme 32:**
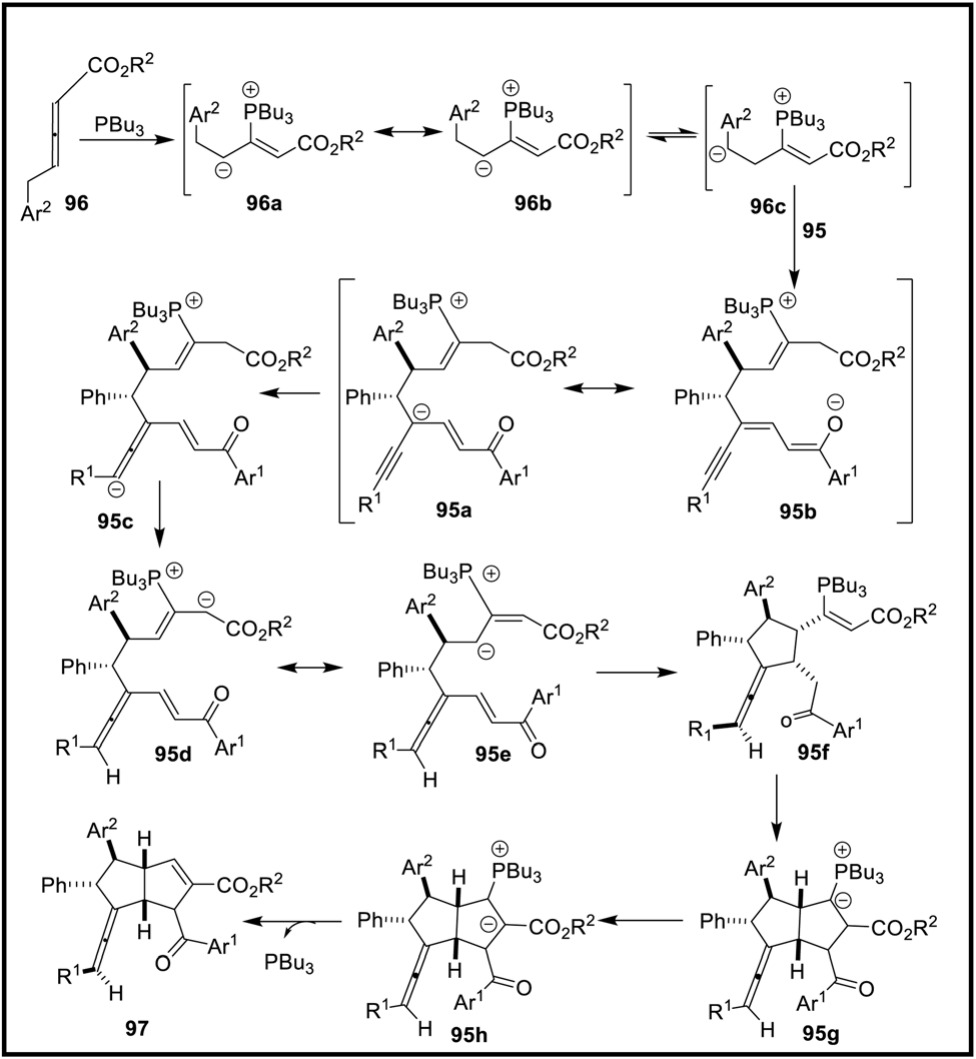
Mechanistic rationale for the [4 + 2] annulation of enynes with γ-substituted allenoates.

The proposed mechanism, illustrated in Scheme 32, begins with the *in situ* generation of ylide intermediate 96b, which undergoes proton migration to furnish zwitterionic intermediate 96c. A subsequent 1,6-conjugate addition to enyne 95 followed by isomerization produces allene intermediate 95c. Sequential proton migrations and annulation events lead to intermediate 95g, and a final proton migration coupled with phosphine elimination delivers the hexahydropentalene product 97. The pronounced diastereoselectivity is rationalized by steric factors, wherein the aryl substituent R^1^ remains spatially distant from the benzoyl group throughout the annulation cascade.

**Scheme 33:**
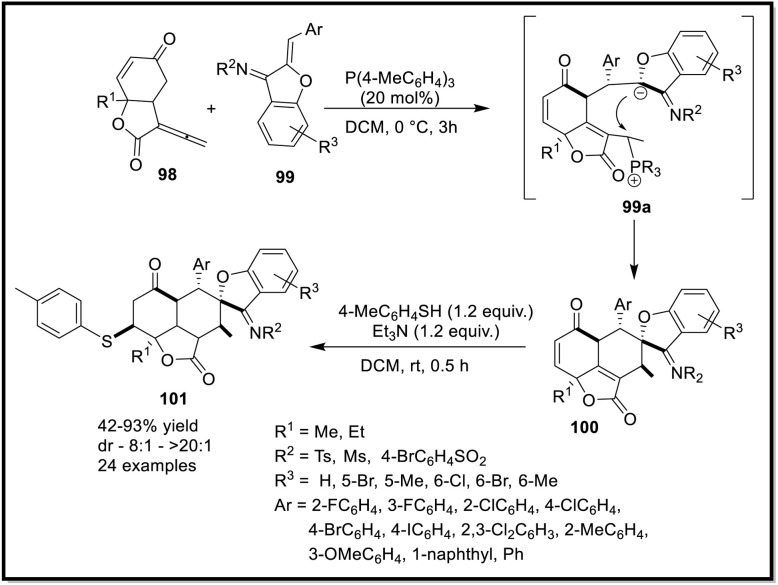
Sequential [4 + 2] annulation involving thio-Michael addition reaction of benzofuranone-derived allenoate with azadiene.

In 2023, Shi *et al.* reported a sequential [4 + 2] annulation/thiomichael addition of benzofuranone-derived allenoate 98 with azadiene 99, affording spirobenzofuran-cyclohexane adduct 100 (Scheme 33).^55^ A phosphine catalyst featuring a toluene substituent efficiently promoted the annulation. Due to characterization challenges of 100, the derivatives were converted into thio-Michael addition products 101 using *p*-methylthiophenol. The protocol tolerated a broad range of substrates, though yields decreased with increasing halogen size. Mechanistically, the reaction initiates *via* nucleophilic phosphine attack on allenoate 98 to form a zwitterionic intermediate, which undergoes two sequential 1,4-hydrogen shifts before attacking azadiene 99 to generate intermediate 99a. Subsequent intramolecular nucleophilic substitution and catalyst regeneration furnish the spirocyclic product 100.

**Scheme 34:**
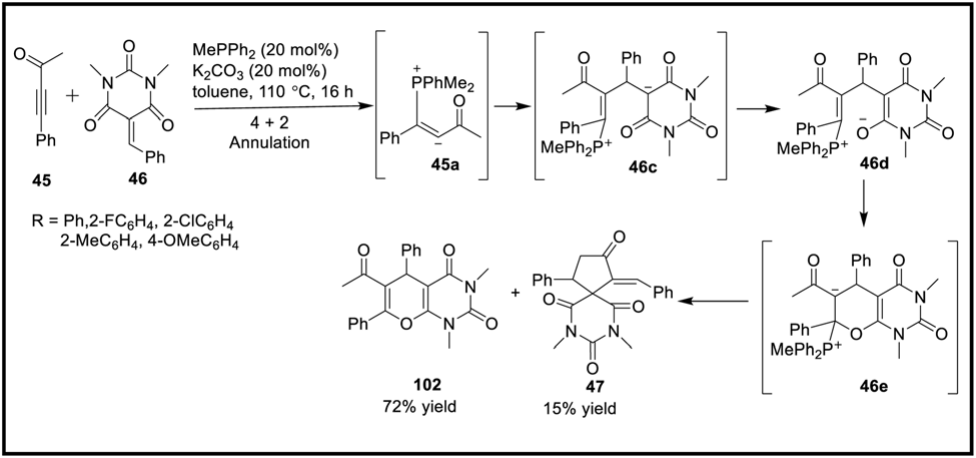
Cascade [4 + 2] annulation of ynones with barbiturate-derived alkenes.

An unprecedented [4 + 2] annulation was reported by Gao and coworkers (Section 2, Scheme 15).^34^ They demonstrated that the choice of base critically influences the annulation outcome of ynone 45 with barbiturate-derived alkene 46, directing the reaction from the conventional [3 + 2] pathway toward a [4 + 2] process, affording product 102 in good yield (Scheme 34). In contrast to the [3 + 2] route, the [4 + 2] annulation involves base-mediated stabilization of intermediate 45a, facilitating direct conjugate addition of alkene 46. This is followed by enolization and intramolecular oxa-Michael addition to generate intermediate 46b. Subsequent cyclization and catalyst regeneration furnish the [4 + 2] adduct 102. Notably, use of Na_2_CO_3_ led to diminished [4 + 2] yield (48%), with the [3 + 2] adduct forming in 51% yield, highlighting the crucial role of base selection in controlling reaction chemoselectivity.

**Scheme 35:**
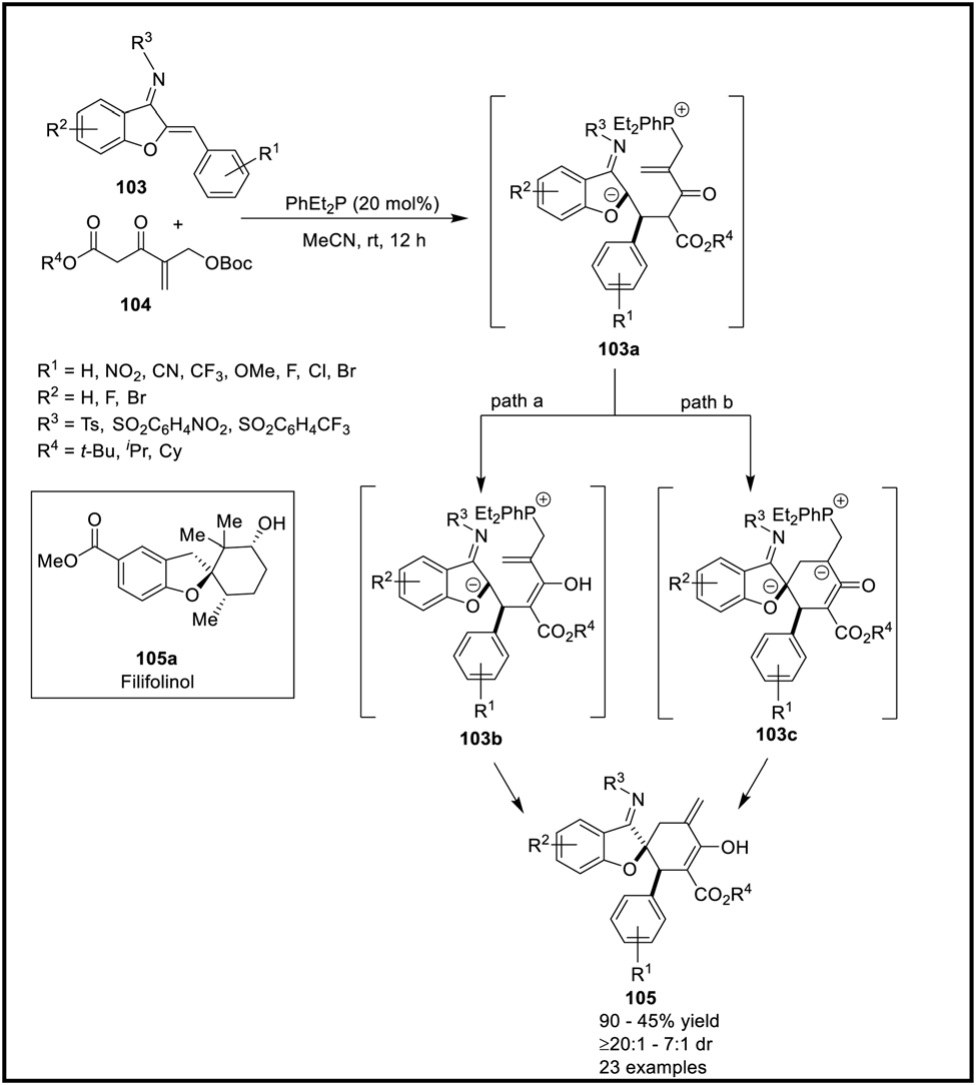
Cascade [2 + 4] annulation of benzofuran-derived azadienes with allyl carbonate.

In 2023, Huang *et al.* reported a novel phosphine-catalyzed [2 + 4] annulation to construct spirobenzofuran-cyclohexane scaffolds 105 featuring two consecutive stereogenic centers (Scheme 35).^56^ This highly regio- and diastereoselective protocol proceeds *via* cyclization of benzofuran-derived azadienes 103 with allyl carbonate 104, affording the product in moderate to excellent yields. The reaction is initiated by nucleophilic substitution of the phosphine catalyst with 104, followed by deprotonation and attack by 103 to generate intermediate 103a. Two mechanistic pathways are plausible: Path a involves enolization of 103a to form intermediate 103b, followed by intramolecular nucleophilic addition to furnish 105; alternatively, an intramolecular Michael addition followed by phosphine elimination and subsequent enolization also leads to the product 105. Substrate scope studies revealed broad tolerance, with azadienes bearing electron-donating, electron-withdrawing, and neutral phenyl substituents affording spirobenzofuran derivatives with excellent diastereoselectivity. This strategy could be further extended to synthesize spirobenzofuran-cyclohexane core-containing natural products, such as filifolinol, an antiviral molecule structurally analogous to 105. Organophosphine-catalyzed [4 + 2] annulation reactions have thus been applied to a broad range of substrates, highlighting their versatility in constructing diverse six-membered cyclic frameworks.

## 6. Asymmetric cascade reactions involving miscellaneous partners: [*a* + *b*]/[*b* + *a*], [*a* + *b*]/[*a* + *c*], [*a* + *a* + *a*]/[*a* + *b*], [*a* + *b*]/[*a* + *b*] and [8 + 2] annulation

Nucleophilic phosphine-catalyzed tandem annulations of allenoates, from unsubstituted to γ-vinyl derivatives, provide efficient access to cyclic and bicyclic frameworks, though the precise orchestration of chemo-, regio-, and diastereoselectivity remains challenging due to multiple reactive sites.

**Scheme 36:**
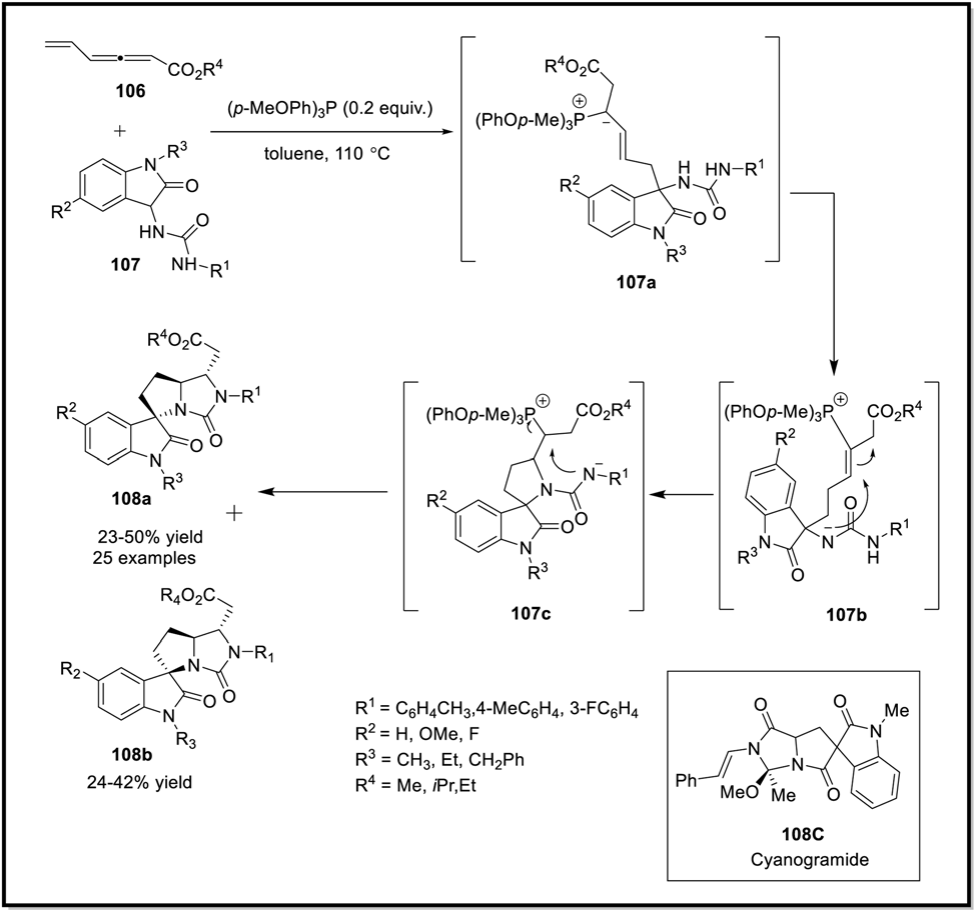
Cascade [8 + 2] annulation of heptafulvene with allenoates.

In 2019, Feng *et al.* reported a phosphine-catalyzed sequential [3 + 2]/[2 + 3] annulation (Scheme 36),^57^ wherein nucleophilic attack of the phosphine catalyst on 106 generates a zwitterionic intermediate that deprotonates urea 107, forming 107a, which undergoes isomerization and hydrogen shift to 107b, followed by intramolecular nucleophilic addition and a second hydrogen shift to 107c, and finally a third nucleophilic substitution with phosphine elimination furnishes the diastereomeric N-hetero-bicyclic hydropyrroloimidazolones 108a and 108b, structurally reminiscent of spiro indole alkaloid 108C, which displays activity against adriamycin- and vincristine-resistant cancer cell lines.

**Scheme 37:**
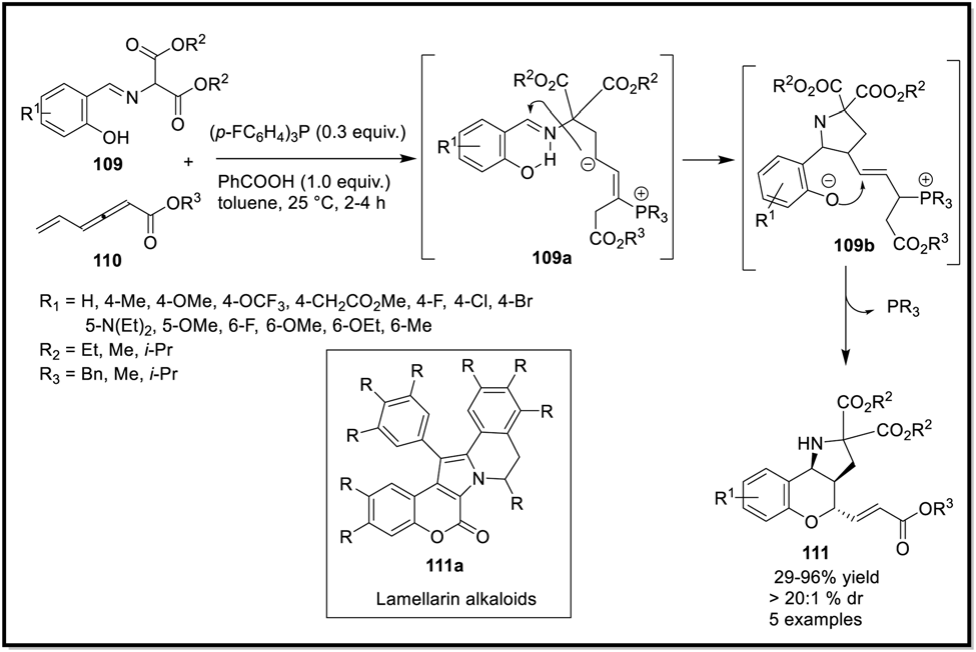
Cascade [3 + 2]/[2 + 3] annulation of allenoate with indoles.

The same group in 2021 further extended the scope of tandem annulation reactions by developing a one-pot tandem [2 + 3]/[2 + 4] annulation reaction of γ-vinyl allenoates 110 with aldimines 109 to achieve chromeno[4,3-*b*]pyrrole derivatives 111 (Scheme 37).^58^ This facile and mild protocol provides access to a wide range of chromenopyrrole derivatives with good yields and remarkable chemo- and diastereoselectivity. However, aldimine containing trisubstituted boron atoms resulted in a lower yield, which slightly improved upon the addition of a PhCOONa/PhCOOH buffer—likely due to PhCOONa coordinating with the boron atom, thus decreasing its nucleophilicity. The phosphine attacks the electron-deficient carbon of the allenoate, forming a zwitterionic intermediate that undergoes conjugate addition with 109 to produce intermediate 109a. This intermediate is resonance-stabilized and further activated by intramolecular hydrogen bonding. Subsequently, the intramolecular carbanion addition of 109a yields intermediate 109b, which then undergoes an intramolecular formal [2 + 4] cycloaddition to afford the product 111, while regenerating the phosphine catalyst. This approach could be further modified for the construction of lamellarins 111a, a family of marine alkaloids, which have been found to inhibit HIV-1 integrase and topoisomerase I.

**Scheme 38:**
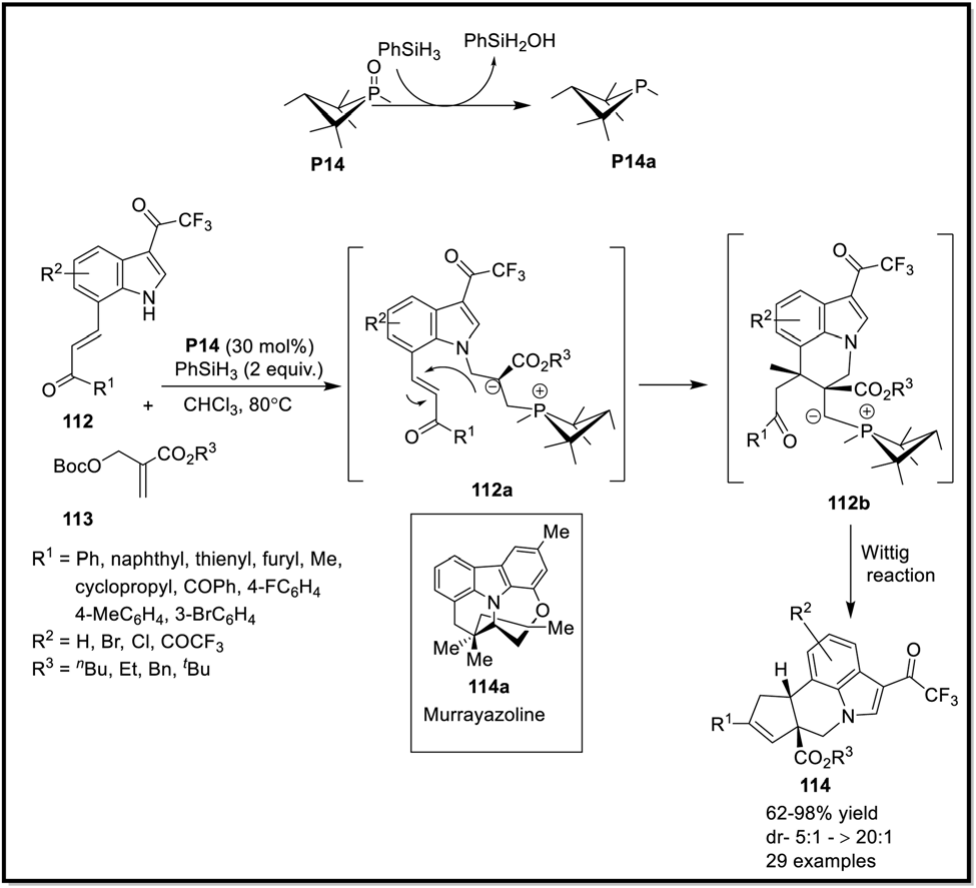
Tandem [2 + 3]/[2 + 4] annulation reaction of γ-vinyl allenoates with aldimines.

Later in 2022, the group reported a tandem [2 + 4]/[2 + 3] annulation strategy for the diastereoselective construction of the pyrroloquinoline scaffold 114 (Scheme 38).^59^ N-protected indoles 112 underwent reaction with MBH carbonate 113*via* two complementary approaches: a stoichiometric PCy_3_-mediated process and a phosphine-catalyzed pathway employing P14. The latter proceeds through a P^III^/P^V^=O catalytic cycle, with P14 serving as the active catalyst. The Michael acceptor 112 displayed broad tolerance toward electron-withdrawing and electron-donating substituents, as well as alkyl-, aryl-, and thienyl-substituted derivatives, furnishing 114 in moderate to good yields with moderate diastereoselectivity. Mechanistically, nucleophilic attack of P14a on 113 generates a ylide intermediate, which undergoes allylic alkylation with 112. Subsequent engagement of P14a affords intermediate 112a, which, following intramolecular Michael addition and proton transfer, yields intermediate 112b. Intermediate 112b then undergoes an intramolecular Wittig cyclization to furnish the product 114. Notably, the adduct 114 displays structural similarity to the carbazole alkaloid murrayazoline, which exhibits antiplatelet aggregation activity. This organophosphine-catalyzed strategy holds significant potential for the synthesis of structurally related polycyclic natural products through judicious modification of indole substrates.

**Scheme 39:**
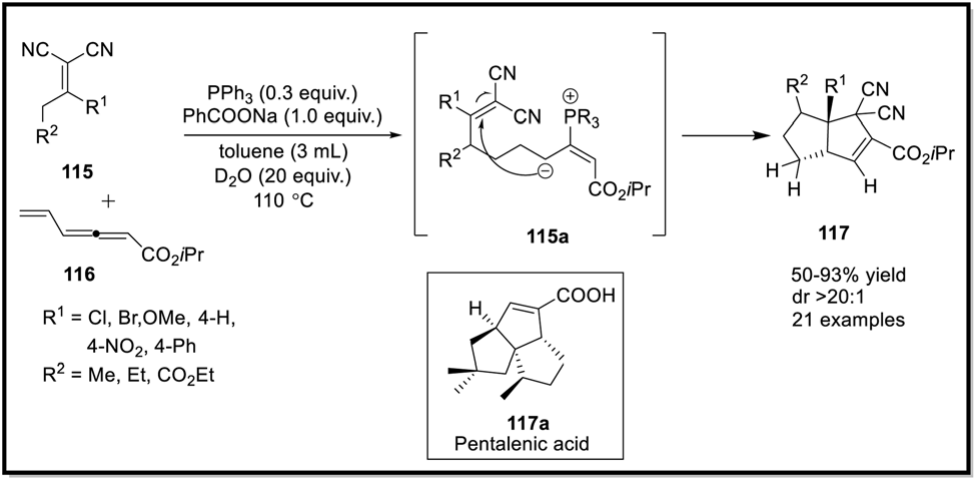
Tandem [2 + 4]/[2 + 3] annulation of N-protected indoles with MBH carbonate.

Feng *et al.* exploited γ-vinyl allenoates as C3 synthons, wherein the conjugated double bonds enable a sequential annulation process (Scheme 39).^60^ This concept culminated in the development of a one-pot phosphine-catalyzed [3 + 2]/[3 + 2] sequential annulation strategy. Under triphenylphosphine catalysis, alkylidenemalononitriles 115 react with γ-vinyl allenoates 116 to afford bicyclic [3,3,0]octene adducts 117 in good to excellent yields. Mechanistically, nucleophilic addition of the phosphine to γ-vinyl allenoate 116 generates a zwitterionic intermediate 115a, which undergoes a 1,7-addition followed by sequential 1,4-addition and an umpolung-type addition, ultimately delivering carbocyclic product 117 bearing a quaternary stereocenter. Notably, this strategy demonstrates broad synthetic versatility, enabling efficient access to a range of fused carbocyclic frameworks, including tri-, tetra-, and pentacyclic skeletons. Moreover, adduct 117 exhibits close structural resemblance to pentalenic acid 117a, a key intermediate in the biosynthetic pathway toward the antibiotic pentalenolactone.

**Scheme 40:**
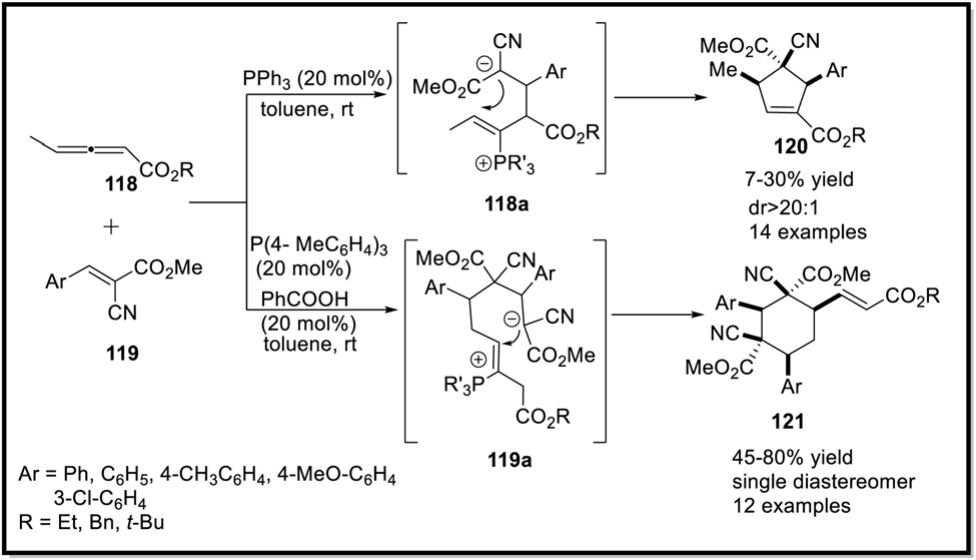
Cascade [3 + 2]/[3 + 2] annulation of alkylidenemalononitriles with γ–vinyl allenoates.

Liu and co-workers reported a highly chemo- and diastereoselective phosphine-catalyzed annulation between γ-methyl allenoates 118 and doubly activated olefins 119, enabling divergent access to either [2 + 2 + 2] or [3 + 2] cycloadducts through acid-controlled pathway selection (Scheme 40).^61^ In the presence of a catalytic acidic additive such as benzoic acid, the reaction proceeds predominantly *via* a [2 + 2 + 2] annulation to furnish polysubstituted cyclohexanes 121 bearing five contiguous stereocenters in high yields with exclusive diastereoselectivity (dr > 20 : 1). In contrast, omission of the acidic additive switches the reaction pathway to an exclusive phosphine-catalyzed [3 + 2] annulation, delivering polysubstituted cyclopentenes 120 containing three contiguous stereocenters in moderate to excellent yields with excellent diastereoselectivity. Mechanistically, nucleophilic attack of the phosphine catalyst on allenoate 118 generates a zwitterionic intermediate that directly adds to activated olefin 119 in the absence of acid, forming intermediate 118a; subsequent cyclization, proton transfer, and phosphine elimination afford the [3 + 2] annulation product 120. By contrast, under acidic conditions, stepwise proton transfer promotes the formation of an allylic phosphorus ylide, which undergoes tandem addition with two equivalents of alkene 119 to generate intermediate 119a; subsequent cyclization, proton transfer, and catalyst elimination furnish the [2 + 2 + 2] annulation product 121.

**Scheme 41:**
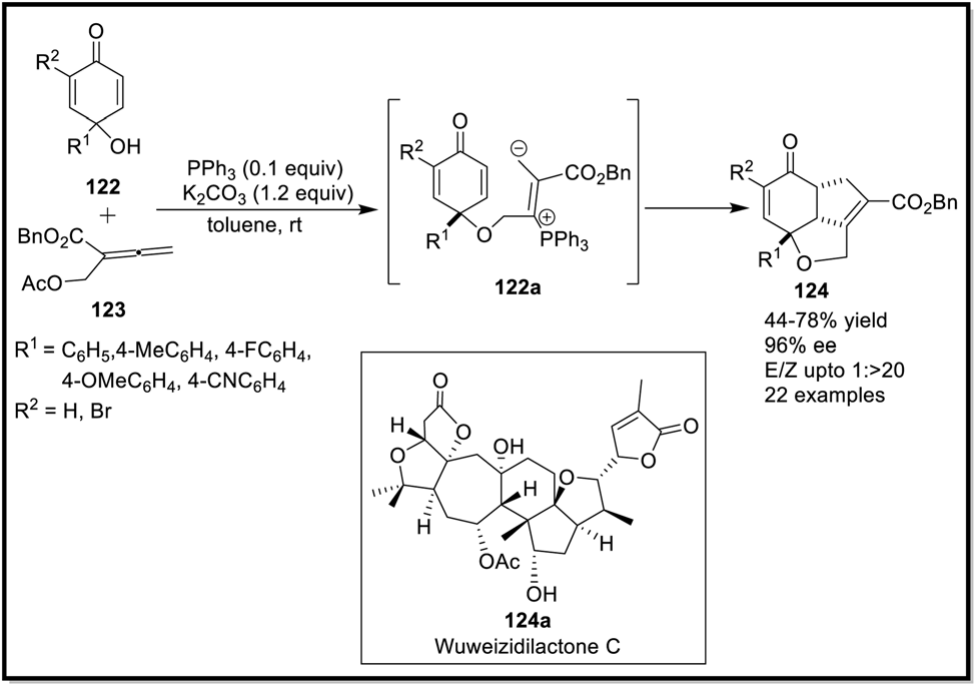
Cascade [2 + 2 + 2] and [3 + 2] annulation of γ-methyl allenoates with doubly activated olefins.

In 2018, Xing *et al.* reported a phosphine-catalyzed cascade annulation strategy for the construction of highly fused hexahydroindenofuran frameworks 124 from *p*-quinols 122 and β′-acetoxy-2,3-butadienoate 123 (Scheme 41).^62^ This protocol exhibits a broad substrate scope, tolerating *p*-quinols bearing aryl, alkyl, and heteroaromatic substituents, and delivers the cascade annulation products in good to excellent yields with high diastereoselectivity. An asymmetric variant was also demonstrated using Kwon's chiral phosphine catalyst, affording product 124 in 39% yield with 55% ee. Mechanistically, β-addition of the phosphine catalyst to allenoate 123 generates a zwitterionic intermediate, which subsequently attacks the deprotonated *p*-quinol to form intermediate 122a. This intermediate undergoes two sequential intramolecular cycloaddition events, followed by phosphine elimination, to furnish the polycyclic product 124. Notably, the resulting ring-fused tetrahydrofuran architecture closely resembles that of the natural product wuweizidilactone C 124a, isolated from *Schisandra chinensis*, which is known to exhibit antiviral activity.^63^

**Scheme 42:**
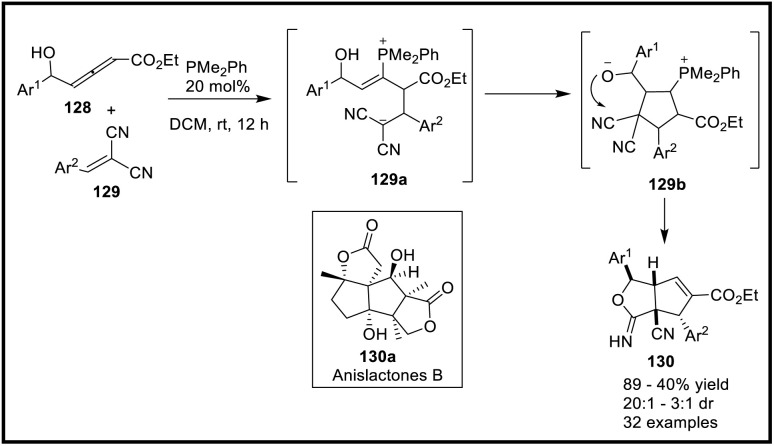
Cascade annulation of *p*-quinoles with β′-acetoxyl-2,3-butadienoate (2018).

In 2023, Duan *et al.* disclosed a phosphine-catalyzed sequential [3 + 2] annulation of allenylic alcohols 128 with dicyanoalkenes 129, enabling the efficient construction of cyclopentafuran derivatives 130 (Scheme 42).^63^ Substrate scope investigations revealed that sterically congested 2-malononitriles 129 markedly enhanced diastereoselectivity, while electron-donating substituents at the *ortho*-, *meta*-, and *para*-positions of the aryl ring were well tolerated and delivered the products in good yields; in contrast, electron-withdrawing groups led to a modest reduction in efficiency. Notably, variation in the electronic nature and substitution pattern of the allenylic alcohol 128 had little influence on the reaction outcome. Mechanistically, the transformation is initiated by nucleophilic phosphine addition to the allenoate moiety, followed by conjugate addition of dicyanoalkene 129 to generate intermediate 129a. Subsequent annulation and proton transfer furnish intermediate 129b, which undergoes an unusual cyclization sequence involving tandem proton shifts and phosphine elimination to afford the bicyclic tetrahydrocyclopentafuran product 130. Importantly, compound 130 exhibits close structural resemblance to the bioactive sesquiterpenoid natural product anislactone B 130a, a known HBV inhibitor. Thus this potential strategy could be extended for the synthesis of cyclopenta[*c*]furan core-embedded natural products.

**Scheme 43:**
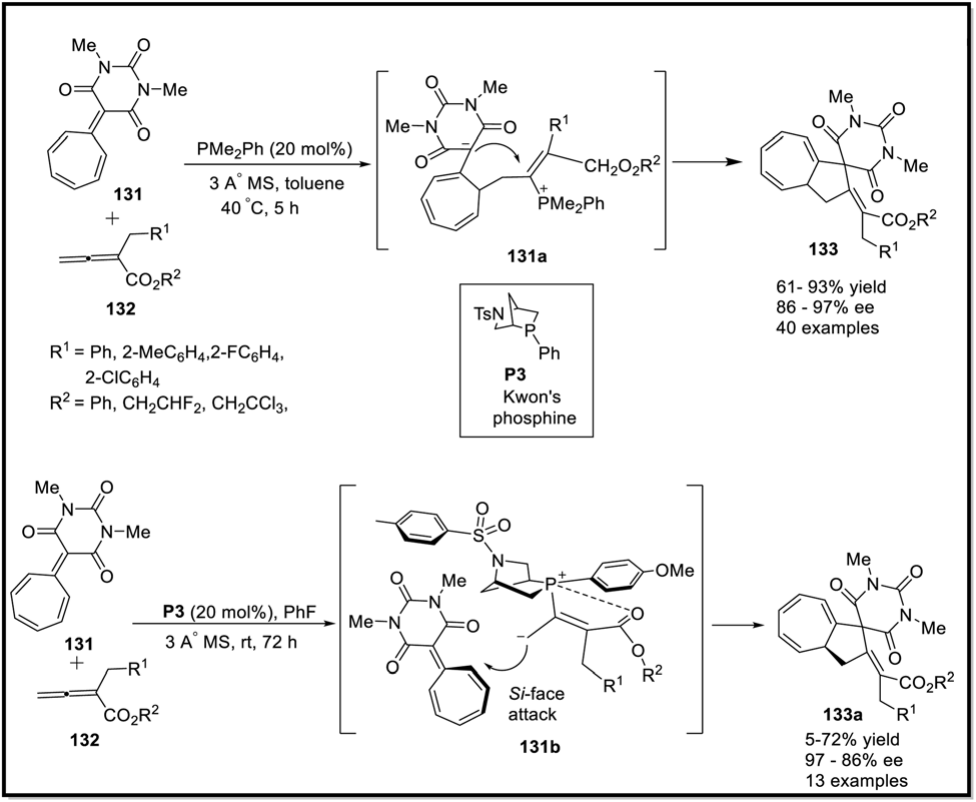
Sequential [3 + 2] annulation reaction of allenylic alcohol with dicyanoalkene (2023).

Higher order annulation reactions represent a powerful strategy for the construction of complex polycyclic frameworks. In 2018, Gao *et al.* reported the first phosphine-catalyzed [8 + 2] annulation of heptafulvene 131 with allenoates 132 under mild conditions, furnishing bicyclo[5.3.0]decane scaffolds 133 (Scheme 43).^64^ The reaction exhibited broad substrate tolerance, accommodating electron-rich, neutral, and electron-deficient aryl substituents, although α-methyl allenoate and ethyl buta-2,3-dienoate were incompatible. The authors also demonstrated an asymmetric variant using Kwon's chiral phosphine catalyst P3, achieving high yields and excellent enantioselectivity. Mechanistically, nucleophilic conjugate addition of the phosphine catalyst to 132 generates a β-phosphonium dienolate intermediate, which attacks 131 to form intermediate 131a. Subsequent intramolecular nucleophilic addition followed by phosphine elimination affords the bicyclic product 133. In the asymmetric variant, *si*-face attack of the phosphonium intermediate on 131b leads to the (*S*)-tricyclic adduct 133a.

Integrating multiple annulation modes within a single reaction manifold remains intrinsically challenging due to the stringent demands of reactivity orchestration and selectivity control; in this regard, γ-vinyl allenoates have emerged as uniquely enabling substrates for tandem annulation strategies, owing to their inherent π-conjugation and programmable reactivity. In addition, only a single report of a phosphine-catalyzed [8 + 2] annulation reaction has appeared in recent years, highlighting significant scope for further exploration in this area.

A rational design of phosphine-catalyzed cascade annulations begins with the identification of the annulation topology, followed by an assessment of the electronic characteristics of the phosphine-derived zwitterionic intermediate. Consideration of steric effects and potential non-covalent interactions can then provide insight into the preferred reaction pathway and stereochemical outcome. Collectively, these factors serve as key parameters for the selection of an appropriate phosphine catalyst and the attainment of the desired diastereo- and enantioselectivity. A summary of the chiral phosphine catalysts discussed in this review is given in Table 1.

**Table 1:** Summary of chiral phosphine catalysts

Sl. No	Chiral phosphine catalyst	Possible stereo-determining effect	ee window (%)
**Annulation topology: [3 + 2]**			
1	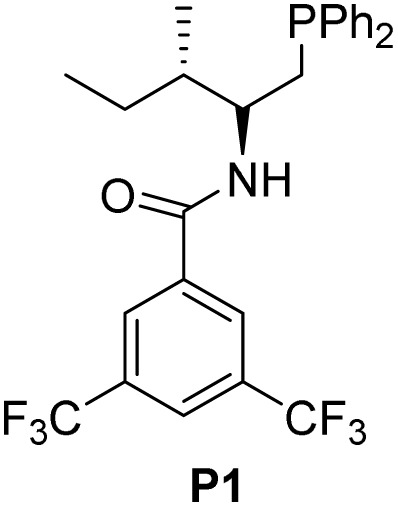	Steric effect	90–95
2	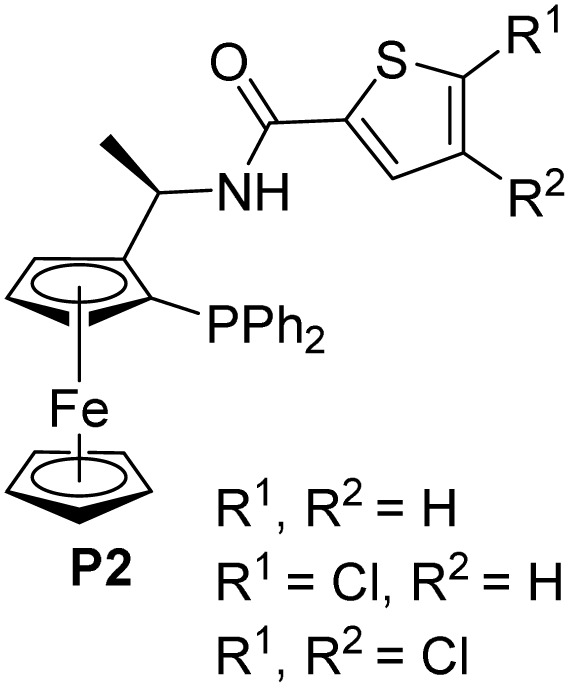	Planar chirality, steric effect	68–77
3	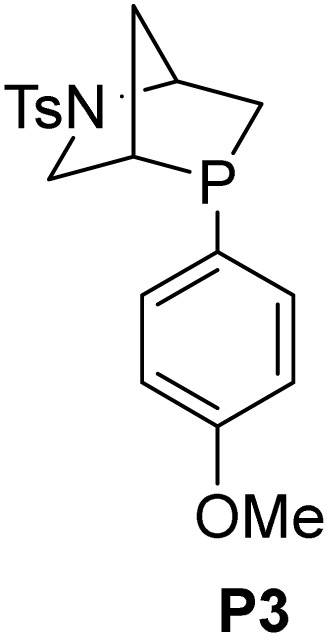	Steric effect	57–99
4	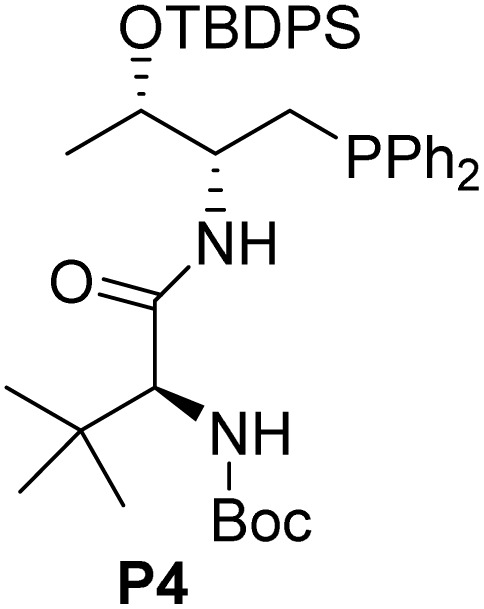	Precise inter and intra molecular H-bonding	74–92
5	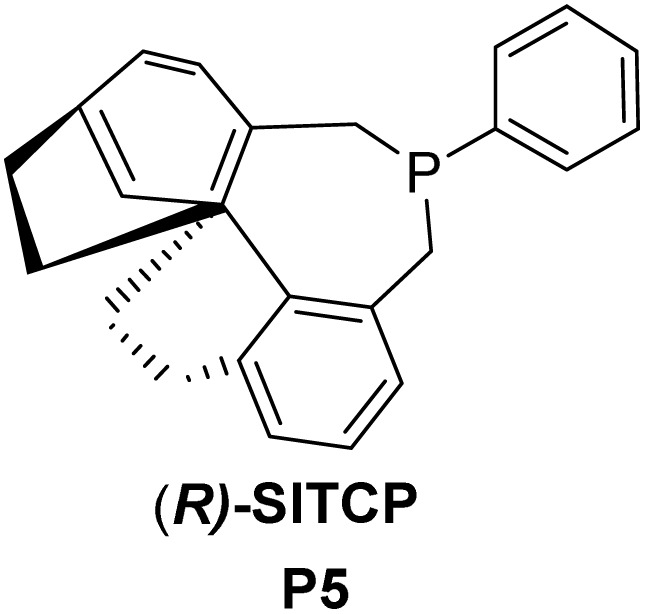	Steric effect	11
6	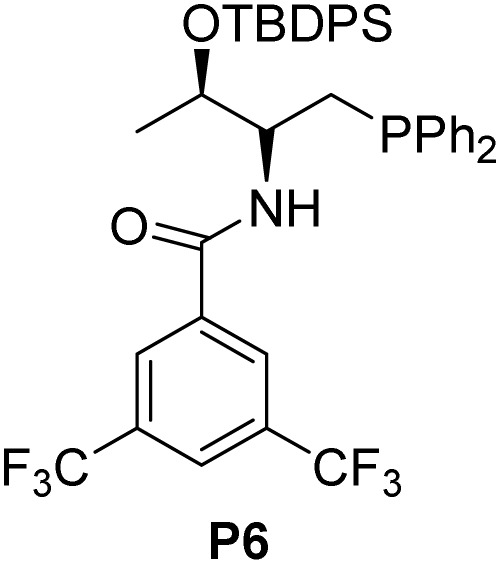	Steric and electronic effect	75–92
7	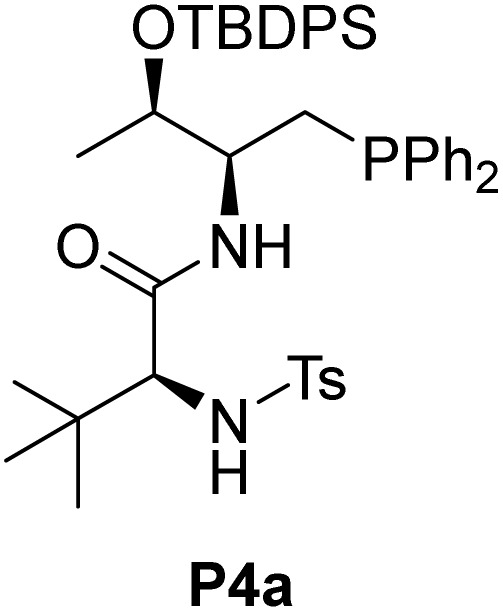	Steric effect	93–82
**Annulation topology: [3 + 3]**			
8	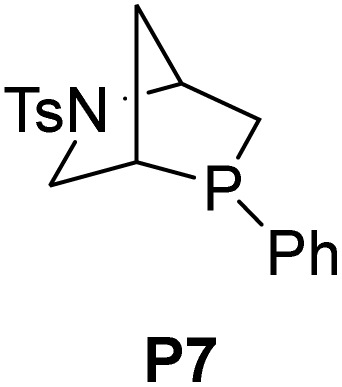	Steric effect	89–20
**Annulation topology: [4 + 1]**			
9	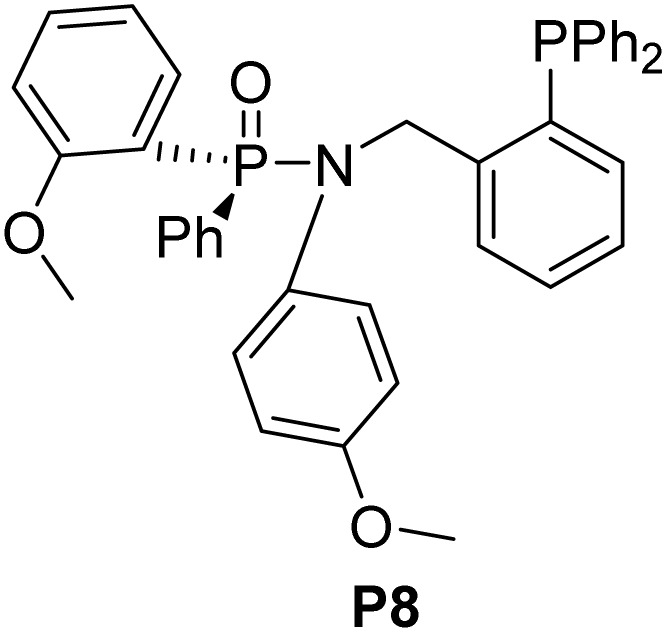	Intramolecular coulombic interactions	56–6
**Annulation topology: [4 + 2]**			
10	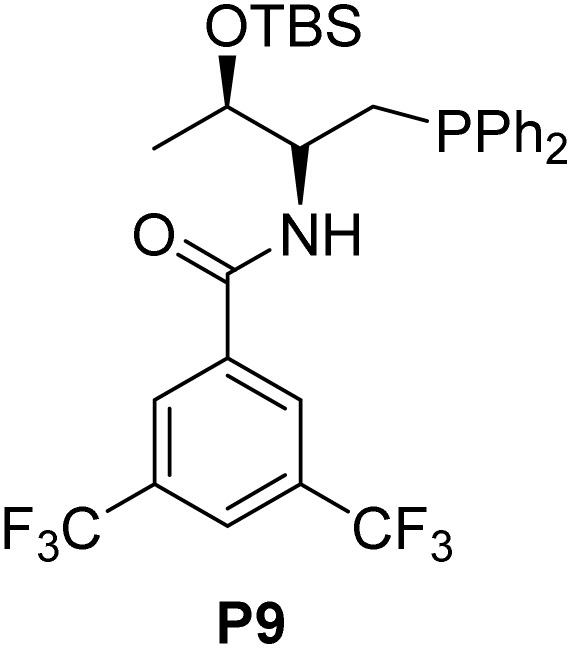	H-bonding	90–94
**Annulation topology: [8 + 2]**			
11	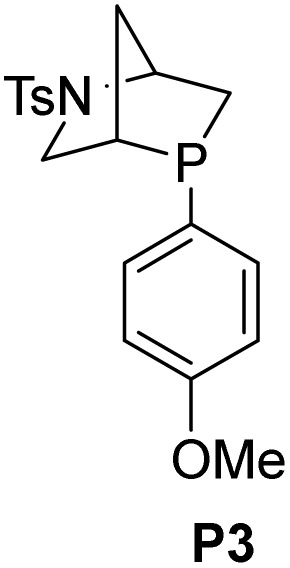	Coulombic interaction, steric effect	97–86

## 7. Summary and future outlook

Phosphine organocatalysis has emerged as a fundamentally distinctive paradigm in asymmetric synthesis, uniquely positioned at the intersection of nucleophilic activation, zwitterionic reactivity, and catalyst-controlled stereochemical control. Across the annulation manifolds surveyed in this Review, phosphine catalysts emerge as molecular linchpins that govern reaction topology, stereochemical evolution, and bond-forming sequences through precise control of orbital alignment and stereoelectronic effects. When viewed through the lens of annulation topology, these transformations reveal a coherent mechanistic framework that unifies diverse reactivity patterns and underscores the exceptional capacity of phosphine catalysis to deliver densely functionalized carbocyclic and heterocyclic architectures under mild conditions.

This review encompasses phosphine-catalyzed annulations, organized according to [3 + 2], [3 + 3], [4 + 1], [4 + 2], and miscellaneous annulation topologies. Each mechanistic manifold is contextualized by its capacity to forge natural-product-inspired architectures and pharmacologically relevant scaffolds, thereby elucidating the central role of phosphine catalysts in steering stereoselective complexity generation through cascade bond-forming events. Future advances in this arena will hinge on attaining predictive control over non-canonical asymmetric induction across extended, multistep reaction sequences. Moreover, higher-order annulation manifolds such as [8 + 2] remain largely underexplored, presenting compelling opportunities for the assembly of architecturally complex carbocyclic and heterocyclic frameworks. Notably, reports on asymmetric induction in chiral phosphine-catalyzed [3 + 3] and [4 + 1] annulations are still scarce, underscoring a significant opportunity for methodological advancement in stereoselective bond construction. The integration of computational methodologies, particularly DFT-guided mechanistic elucidation and catalyst–substrate interaction analysis, is poised to enable the rational design of next-generation phosphine catalysts with enhanced reactivity and stereocontrol. Looking forward, the integration of continuous-flow technology with phosphine-catalyzed asymmetric synthesis may offer new opportunities to enhance process scalability and sustainability, ultimately promoting the industrial adoption of these catalytic transformations.

Future breakthroughs in phosphine organocatalysis will be driven by the synergistic integration of mechanistic insight with the rational design of next-generation chiral phosphines capable of enforcing stereochemical fidelity across multiple, sequential bond-forming events. A nuanced understanding of zwitterionic intermediate lifetimes, catalyst–substrate recognition, and cooperative noncovalent interactions will be pivotal to translating these conceptual advances into broadly applicable, predictable synthetic strategies. Collectively, these efforts are poised to elevate phosphine-catalyzed annulations from powerful methodological tools to a central, generalizable platform for the enantioselective construction of architecturally intricate carbocyclic and heterocyclic frameworks, with profound implications for natural product synthesis, medicinal chemistry, and molecular innovation. By unifying mechanistic paradigms in representative cascade cyclizations, and illustrating their utility in high-value synthetic applications, especially in natural product and hybrid molecular synthesis; this work redefines phosphine organocatalysis as a strategic, stereocontrolled engine for rapid molecular complexity generation rather than a mere collection of isolated reactions. This review closes the key gaps arising from fragmented understanding of stereocontrol and reactivity. Distinct from prior reviews, this review systematically examines the pivotal role of phosphine catalysts as linchpins in enabling non-canonical asymmetric induction during cascade cyclizations.

## Author contributions

Chithra Mohan: conceptualization, supervision, and critical review and editing of the manuscript. R. Bharath Krishna: conceptualization, drafting, supervision, critical review and editing of the manuscript. Archana Vijayakumar: writing – original draft, preparation of schematic illustrations. Manod M: writing – original draft, preparation of schematic illustrations. Aditya Appus: assistance in initial drafting of the manuscript.

## Conflicts of interest

There are no conflicts to declare.

## Data Availability

No new data were created or analyzed in this study.
